# Influence of modelling disorder on Hirshfeld atom refinement results of an organo-gold(I) compound

**DOI:** 10.1107/S2052252522005309

**Published:** 2022-06-11

**Authors:** Sylwia Pawlędzio, Maura Malinska, Florian Kleemiss, Simon Grabowsky, Krzysztof Woźniak

**Affiliations:** a Biological and Chemical Research Centre, Department of Chemistry, University of Warsaw, Żwirki i Wigury 101, Warsaw 02-089, Poland; b Faculty for Chemistry und Pharmacy, University of Regensburg, Universitätsstraße 31, 93053 Regensburg, Germany; c Department of Chemistry, Biochemistry and Pharmaceutical Sciences, University of Bern, Freiestrasse 3, 3012 Bern, Switzerland

**Keywords:** disorder, Hirshfeld atom refinement, heavy elements, relativistic effects, quantum crystallography

## Abstract

Details of the validation of disorder modelling with Hirshfeld atom refinement (HAR) for a previously investigated organo-gold(I) compound are presented here. The impact of refining disorder on HAR results is discussed using an analysis of the differences of dynamic structure factors. These dynamic structure factor differences are calculated from thermally smeared quantum mechanical electron densities based on wavefunctions that include or exclude electron correlation and relativistic effects. When disorder is modelled, the electron densities stem from a weighted superposition of two (or more) different conformers. Here this is shown to impact the relative importance of electron correlation and relativistic effect estimates expressed by the structure factor magnitudes. The role of disorder modelling is also compared with the effect of the treatment of hydrogen anisotropic displacement parameter (ADP) values and atomic anharmonicity of the gold atom. The analysis of ADP values of gold and disordered carbon atoms showed that the effect of disorder significantly altered carbon ADP values and did not influence those of the gold atom.

## Abstract 2

Incorporation of the description of disorder during Hirshfeld atom refinement influences the distribution of dynamic structure factors and, in consequence, also the contributions from electron correlation and relativistics.

## 1. Introduction

The number of structures deposited in the Cambridge Structural Database (CSD) is increasing quickly and has surpassed one million structures (Groom *et al.*, 2016 ▸), almost 30% of which are disordered (Groom *et al.*, 2016 ▸). Generally, disorder occurs when atoms, functional groups or even whole molecules are located in different positions in different unit cells in the crystal (Müller *et al.*, 2006 ▸). Usually, disorder occurs in only some parts of the molecule, such as freely rotating methyl (Kaiser Morris *et al.*, 1997 ▸; Roessler *et al.*, 2000 ▸) or *tert*-butyl groups, solvent molecules (Teeter, 1992 ▸), and other organic functional groups or long side chains (Inoue *et al.*, 2022 ▸). Detecting and modelling disorder is important not only in crystallography (Dittrich, 2021 ▸), but also in other scientific fields, including materials chemistry (Varn & Crutchfield, 2015 ▸), the pharmaceutical industry (Dittrich, 2021 ▸) or the study of protein functions (Atkins *et al.*, 2015 ▸). For example, it has been reported that disordered graphene nanosheets have very high reversible capacities and are promising candidates for high-capacity Li ion batteries (Pan *et al.*, 2009 ▸). Also, disordered proteins have been found to play an important role in DNA binding and signalling cascades (Atkins *et al.*, 2015 ▸).

In crystallography, the refinement of disorder involves occupancy parameters for the different positions in the unit cell, and is most conveniently performed with *SHELXL* (Sheldrick, 2008 ▸, 2015 ▸) using the graphical interface of *Olex2* (Dolomanov *et al.*, 2009 ▸). Of course, such refinement is also possible with other common crystallographic packages such as *olex2.refine* (Bourhis *et al.*, 2015 ▸), *CRYSTALS* (Betteridge *et al.*, 2003 ▸) or *JANA* (Petříček *et al.*, 2014 ▸) *etc*. So far, the most popular model used for structural refinement with disorder has been the independent atom model (IAM) (Compton, 1915 ▸). In this approach, the atomic scattering factors are obtained from theoretical calculations of spherically averaged atomic electron densities for isolated and uncharged atoms. As a consequence, the information about the aspherical electron density in the bonding and lone pair regions or between interacting molecules is lost. Description of spherical electron density is not sufficient for atoms of heavy elements either, since they are easily polarizable and adopt aspherical shapes when in molecules (Zhurov *et al.*, 2011 ▸). Moreover, the use of the IAM systematically underestimates *X*–H distances (Woińska *et al.*, 2016 ▸) and does not allow for the refinement of hydrogen anisotropic displacement parameters (ADPs). Therefore, the IAM is increasingly being replaced by more sophisticated descriptions of the electron density in crystals (Hansen & Coppens, 1978 ▸; Jayatilaka, 1998 ▸; Jayatilaka & Dittrich, 2008 ▸; Capelli *et al.*, 2014 ▸). The most popular method, which overcomes all the shortcomings of the IAM, is Hirshfeld atom refinement (HAR) (Jayatilaka & Dittrich, 2008 ▸; Capelli *et al.*, 2014 ▸). This is an iterative procedure, where ‘tailor-made’ aspherical atomic scattering factors (Hirshfeld, 1977 ▸), obtained by quantum mechanical methods, are used to refine the atomic positions and ADPs in the standard, least-squares structure refinement until convergence. Previous work reported that HAR provides more accurate hydrogen atom positions (Woińska *et al.*, 2016 ▸, 2021 ▸; Malaspina *et al.*, 2020 ▸) than IAM and enables refinement of hydrogen ADP values (Wanat *et al.*, 2021 ▸). Furthermore, HAR utilizing relativistic Hamiltonians was also successfully applied to heavy elements (Pawlędzio *et al.*, 2021 ▸; Bučinský *et al.*, 2019 ▸, 2016 ▸).

**Figure 1:**
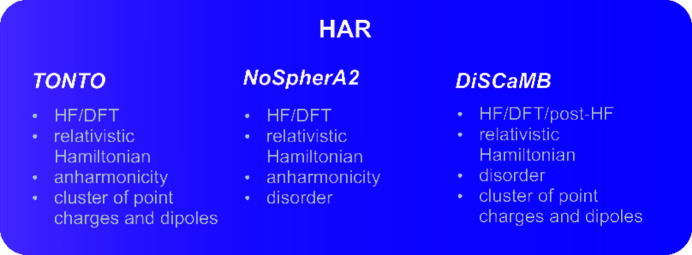
Available software for HAR and their most important features.

Three implementations of HAR are currently available (Fig. 1 ▸). The original HAR (Jayatilaka & Dittrich, 2008 ▸) is implemented in the *Tonto* (Jayatilaka & Grimwood, 2001 ▸) software, and can be used under two graphical interfaces: *lamaGOET* (Malaspina *et al.*, 2021 ▸) and *HARt* in *Olex2* (Fugel *et al.*, 2018 ▸) (only up to version 1.5). *Tonto* enables calculations of structure factors at either Hartee–Fock (HF) (Strinati, 2005 ▸) level of theory or using density functional theory (DFT) (Sholl & Steckel, 2009 ▸) with different functionals such as, for example, BLYP/B3LYP and relativistic Hamiltonians (IOTC and DKH). Also, refinement of anharmonic thermal motions and the simulation of the crystal field by a cluster of point charges and dipoles around the investigated molecule are available (Dittrich *et al.*, 2012 ▸; Woinska *et al.*, 2019 ▸). Unfortunately, HAR cannot be used with this software implementation for disordered and polymeric structures, for structures in non-centrosymmetric space groups with special positions, or for twinned crystals. Moreover, using HAR with *Tonto* is computationally very expensive, and it is not well optimized for atoms heavier than krypton.

One idea to speed up HAR was to integrate it with libraries of extremely localized molecular orbitals (ELMOs; Meyer & Genoni, 2018 ▸). The HAR–ELMO (Malaspina *et al.*, 2019 ▸) method, however, is at present mostly dedicated to the refinement of proteins. To overcome the limitations of *Tonto* and make HAR more user-friendly for small molecules, two independent HAR methods were recently developed (Kleemiss *et al.*, 2021 ▸; Chodkiewicz *et al.*, 2018 ▸, 2020 ▸). Both the *NoSpherA2* (Kleemiss *et al.*, 2021 ▸) and the *DiSCaMB* (Chodkiewicz *et al.*, 2018 ▸) libraries allow the use of HAR with the *Olex2* GUI (Dolomanov *et al.*, 2009 ▸). Single-point calculations can be carried out by well known pieces of quantum chemistry software, such as *ORCA* (Neese, 2012 ▸; Neese *et al.*, 2020 ▸), *GAMESS* (Barca *et al.*, 2020 ▸) or *Gaussian* (Frisch *et al.*, 2016 ▸), which makes a variety of quantum mechanical methods available including the relativistic Hamiltonian. Since HARs in these frameworks use the same refinement engine as the IAMs [namely *olex2.refine* (Bourhis *et al.*, 2015 ▸)], they can be performed with constraints and restraints. The new HARs are performed against *F* ^2^, and a *Shelx*-type weighting scheme can be applied. *NoSpherA2* provides an opportunity for refinement of anharmonic atomic motion (Mallinson *et al.*, 1988 ▸), which might be important for heavier atoms (Pawlędzio *et al.*, 2021 ▸). However, *NoSpherA2* does not permit building up clusters of charges around the central molecule to simulate the crystal field, only solvation models. On the other hand, *DiSCaMB* enables refinements with a simulated crystal field environment, and offers a choice of different electron density partitioning (Chodkiewicz *et al.*, 2020 ▸), as well as the use of post-HF methods (Magnasco, 2013 ▸); however, refinement of anharmonic atomic motion (Mallinson *et al.*, 1988 ▸) is not possible.

Our previous work on relativistic HAR for an organo-gold(I) compound showed that relativistic effects, electron correlation and atomic anharmonicity significantly influence the electron density distribution in the crystal (Pawlędzio *et al.*, 2021 ▸). The detected partial disorder of one phenyl ring was not included, since it was impossible to handle disorder with HAR in the software *Tonto*. Very recently, Kleemiss *et al.* (2021 ▸) proposed how to model disorder in HAR using *NoSpherA2* implemented in *Olex2* (Holsten *et al.*, 2021 ▸). Since then, the HAR method can be applied for structures with a higher degree of complexity of disorder, including cases where heavy atoms are present or for polymeric structures. It is finally possible for quantum crystallography to be employed by a wider range of researchers, especially for poorly diffracting crystals. This is possible due to the advancement of the methodology, in particular models of atomic electron density and the development of software, for example, the recently published work on HAR with periodic DFT calculations with the projector augmented wave method (Ruth *et al.*, 2022 ▸), or new implementations of *DiSCaMB* (Chodkiewicz *et al.*, 2018 ▸) in *CRYSTALS* (Betteridge *et al.*, 2003 ▸); however, in this latter case its related functionality is not yet publicly available.

Here, we decided to reinvestigate our study with *NoSpherA2* (Kleemiss *et al.*, 2021 ▸) because it is the only software that allows us to study disorder and anharmonicity together. We validated the modelling of disorder with HAR using different levels of theory for the theoretical wavefunctions used to produce the non-spherical scattering factors, namely HF and DFT with and without relativistic corrections. This means that both the effects of electron correlation via the DFT functional and the relativistic effects can be studied separately. It was investigated earlier what these differences in the wavefunctions mean for the derived structure factors, and in which domains and regions these effects are more or less pronounced relative to each other (Bučinský *et al.*, 2016 ▸, 2019 ▸; Pawlędzio *et al.*, 2022 ▸). Here, we study whether the relative importance of these two physical effects changes if not one wavefunction is used to calculate the scattering factors and ultimately the structure factors, but two. Two different wavefunctions at two different geometries are needed for the two disorder components of the molecule in the crystal structure (Bourhis *et al.*, 2015 ▸), and the two resulting quantum mechanical wavefunctions are then merged using the weights of the refined disorder occupation/population parameters. More details on how disorder is treated in *NoSpherA2* are discussed in the literature (Kleemiss *et al.*, 2021 ▸). In any case, we can only indirectly assess electron correlation and relativistic effects through changes in the dynamic structure factors since there are no parameters or descriptors in HAR, or the wavefunction itself, for that matter, that measure or represent these two effects. Here, we discuss the changes in the structure factors also in relation to their effect on the refined parameters, namely hydrogen and carbon ADPs and gold anharmonicity.

## 2. Experimental and computations

### 2.1. X-ray data collection

The high-resolution X-ray diffraction experiment was performed using synchrotron radiation at the BL02B1 beamline (SPring-8 synchrotron (SP8), Japan). The X-ray energy used during the experiment was 50 keV (λ = 0.2486 Å). The experiment was carried out at 80 K using a Huber 1/4χ-axis goniometer equipped with a Pilatus3 X 1M CdTe (P3) detector. The Pilatus images were converted to the Bruker .sfrm format and integrated as described in our previous paper (Pawlędzio *et al.*, 2021 ▸).

### 2.2. Structure determination

**Figure 2:**
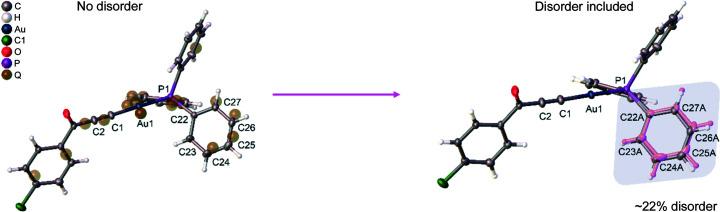
Structure of the [3-(4-chloro­phenyl)-3-oxoprop-1-yn-1-yl](tri­phenyl­phosphine)gold(I) complex measured at 80 K. The phenyl ring shown above is affected by 22% disorder after IAM. The electron density peaks visible in the plane of the ring defined for the C22–C27 atoms (brownish transparent balls) show the second set of atomic positions.

The structure of the compound studied here has been published recently (Pawlędzio *et al.*, 2021 ▸). However, the models did not include the partial disorder (∼22%) of one of the phenyl rings (Fig. 2 ▸). Here, we have reinvestigated the structure of [3-(4-chloro­phenyl)-3-oxoprop-1-yn-1-yl](tri­phenyl­phosphine)gold(I) and modelled the above-mentioned disorder. The initial model was obtained with IAM using *SHELXL* (Sheldrick, 2008 ▸, 2015 ▸) within the graphical interface of *Olex2* (Dolomanov *et al.*, 2009 ▸). To do this, the positions of carbon atoms (C22–C27) that showed suspiciously large ADPs were split into two parts, resulting in two conformations of 78 and 22% occupation, respectively. Also, some restraints were applied for the C23A–C27A atoms to fit the hexagonal shape for the phenyl ring and keep the same values of the corresponding C—C distances from the major and minor components of the disordered parts with a sigma value of 0.02 Å. This initially refined model was then used as an input for HAR. The details of X-ray data collection and structure refinement after IAM with disorder are given in Table S1 of the supporting information.

### 2.3. Hirshfeld atom refinement

HARs were performed with the *NoSpherA2* (Kleemiss *et al.*, 2021 ▸) interface implemented in *Olex2* (Dolomanov *et al.*, 2009 ▸) using full-matrix least-squares in *olex2.refine*. The molecular wavefunctions were calculated with *ORCA* (Neese, 2012 ▸, 2020 ▸) either at the HF (Strinati, 2005 ▸) or at the DFT/PBE (Sholl & Steckel, 2009 ▸) levels of theory, either using the non-relativistic or relativistic second-order Douglas–Kroll–Hess (DKH2) (Reiher, 2012 ▸; Reiher & Wolf, 2004 ▸) Hamiltonian, utilizing Jorge-TZP and Jorge-TZP-DKH basis sets (Martins *et al.*, 2015 ▸; Pritchard *et al.*, 2019 ▸), respectively. Any of these wavefunctions were read by the *NoSpherA2* (Kleemiss *et al.*, 2021 ▸) software; the related electron-density was partitioned into Hirshfeld atoms, whose Fourier transforms are the non-spherical scattering factors, which were then tabulated in a .tsc file and handed over to *olex2.refine* (Bourhis *et al.*, 2015 ▸) for the least-squares refinement. More details on the procedure are discussed by Midgley *et al.* (2021 ▸).

The geometry including disorder modelling after spherical refinement was used as input. For the sake of comparison HARs without disorder modelling were also performed. In all refinements, Δ*f*′ and Δ*f*′′ parameters for the anomalous dispersion correction were taken from the Sasaki table (Sasaki, 1989 ▸). The high integration accuracy setting for the calculations of grids was used, with the normal SCF convergence threshold and a slow convergence SCF strategy.

Positions of hydrogen atoms and their ADPs were refined in two ways. In the first case, the positions of hydrogen atoms and their ADPs were freely refined with the exception of hydrogen atoms from the minor disorder component. These hydrogen atoms were refined isotropically as riding atoms with distances fixed at 1.1 Å, as informed by neutron experiments (Allen & Bruno, 2010 ▸). In the second case, we refined the positions of the hydrogen atoms as described above, but this time the ADP values for hydrogen atoms were estimated using SHADE3 (Madsen, 2006 ▸). In both situations, the restraints, which were applied for the C23A–C27A atoms during IAM, were also included in HAR. All HAR models included anharmonic displacement factors from the Gram–Charlier expansion (Mallinson *et al.*, 1988 ▸) of the ADP values for Au (the third- and fourth-order values of the Gram–Charlier coefficients are listed in Tables S3 and S4). Table 1 ▸ lists all abbreviations used in this study to distinguish HARs. Statistical parameters of all HARs performed are given in Table S2.

## 3. Results and discussion

### 3.1. Refinement comparison

**Figure 3:**
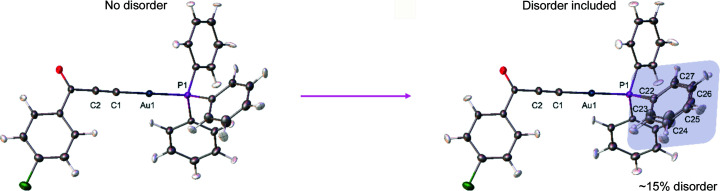
Structure of the investigated compound after HAR refinement (rks_rel_anis). The phenyl ring shown above is affected by 15% disorder after HAR.

Table 2 ▸ shows a comparison of the agreement statistics obtained after the IAM and HAR refinements (rks_rel_anis_dis and rks_rel_anis_no_dis models) with and without disorder, respectively. It is undeniable that when the quality of diffraction data is good enough, more complex models should describe electron density better than simpler models which do not take disorder into account. Usually, this is associated with a lowering of the *R*-values and lowering maxima of the residual electron density. Here, the differences are very small and mostly reflected in the slightly lower *R*-factors (IAM_no_dis *versus* IAM_dis, and rks_rel_anis_no_dis *versus* rks_rel_anis_dis). The situation is similar when looking at rks_rel_shade_no_dis *versus* rks_rel_shade_dis (Table S2). These differences become more visible when comparing IAM and HAR, which is not surprising, since IAM does not describe aspherical features such as electron density of lone pairs or chemical bonds. It is also worth noting that the application of HAR changes the occupancies in the final refined model thus reducing the percentage of disorder from 22 (Fig. 2 ▸) to 15% (Fig. 3 ▸).

**Figure 4:**
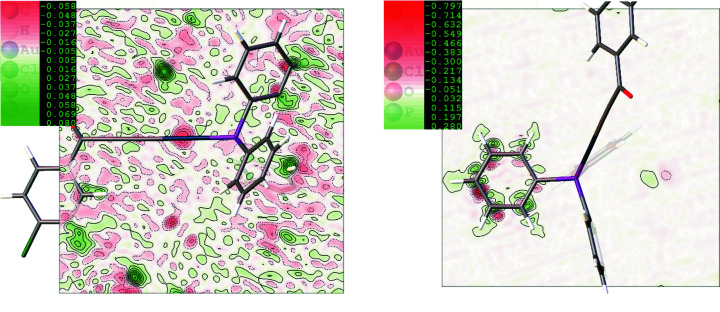
2D visualization of the dynamic electron density (eÅ^−3^) calculated as the difference between rks_rel_anis_dis and rks_rel_anis_no_dis HAR models in the vicinity of (*a*) C–Au–P atoms and (*b*) the disordered phenyl ring. The dynamic electron density was calculated with *Olex2* computed by inverse Fourier transformation of calculated structure factors.

We also show two-dimensional maps of dynamic electron density differences between HAR models: rks_rel_anis_dis and rks_rel_anis_no _dis (Fig. 4 ▸). As can be seen, modelling of the disordered phenyl ring caused only small changes in the vicinity of Au [±0.06 e Å^−3^, Fig. 4 ▸(*a*)]. Naturally, bigger changes were observed in the region of the disordered phenyl ring, with the maximum and minimum values ranging from +0.3 to −0.7 e Å^−3^, respectively [Fig. 4 ▸(*b*)]. The question arises what, if anything, has actually changed in the parameters associated with anharmonicity and in the signatures of relativistic effects and electron correlation? How do they change when disorder is included in the refinement procedure?

To answer this question, we decided to carefully analyse the changes between HAR models and investigate the impact of disorder modelling (DIS) on the obtained ADPs and structure factors. We also compared the DIS effect with effects of treatment of hydrogen ADPs (SHADE) and refinement of atomic anharmonic thermal motion of Au (ANH). Finally, we inspected how the above-mentioned refined parameters are affected by relativistic effects (REL) and electron correlation (ECORR).

### 3.2. Effect of modelling disorder on the ADPs

**Figure 5:**
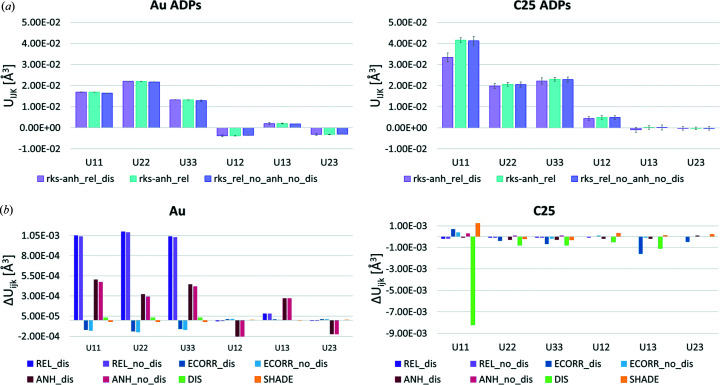
(*a*) Plot of the numerical values of the Au and C25 ADPs (Å^2^) within three e.s.d.’s for models at the rks_rel level of theory with disorder, anharmonicity or no disorder and no anharmonicity; (*b*) effect of REL (rks_anh_rel – rks_anh_nr), ECORR (rks_anh_rel – rhf_anh_rel), ANH (rks_anh_rel – rks_rel) and DIS (rks_anh_rel – rks_anh_rel) in Au and C25 ADPs (Å^2^). For REL, ECORR and ANH the effect of disorder is also shown.

Fig. 5 ▸(*a*) shows a plot of the ADPs of Au and C25 within three estimated standard deviations (e.s.d.’s) for three HAR models: rks_rel_anh_anis_dis, rks_rel_anh_anis and rks_rel_anis. The plot illustrates the impact of modelling disorder and compares it with the effect of modelling the atomic anharmonic thermal motion of Au. It can be seen that the Au ADP values are affected by modelling of the anharmonicity, but are not affected by modelling of the disorder at the rks_rel level of theory. The situation for C25 in the disordered phenyl group is, of course, slightly different. The biggest change is observed for the *U*_11_ element of the ADP tensor, leading to overall smaller ADP values for this carbon atom when disorder was included, but did not change on refinement of anharmonic thermal motion for Au. This indicates that the effects of disorder in the phenyl ring and the description of anharmonicity of Au are not correlated. This was also confirmed in Fig. 5 ▸(*b*), which presents an overview of all the investigated effects and how accounting for disorder influenced the description of these effects. For the Au ADPs, the effects of DIS and SHADE are very small, smaller than three standard uncertainties, and did not significantly change the magnitude of the ADP values related to relativistic effects, electron correlation or anharmonicity.

However, when considering the consequences of including anharmonicity, it influenced HAR refined ADP values related to both relativistic and electron correlation effects and increased these effects by an order of magnitude (Tables S6 and S8). In the case of the C25 ADP, the DIS effect is important for the diagonal *U*_11_, *U*_22_ and *U*_33_ components of the ADP tensor. The differences are larger than three standard uncertainties, with the biggest change observed for the *U*_11_ component [Fig. 5 ▸(*b*) and Table S9]. The effect of SHADE is also significant and influenced the *U*_11_ component, while the effects of accounting for REL, ECORR and ANH for the C25 ADP are negligible [Fig. 5 ▸(*b*) and Table S9].

As reported in our previous work, refined ADP values depend on the level of theory used for HAR including an increase of the Au ADP on inclusion of REL and ECORR in the wavefunction. This observation corresponds well with the displacement obtained in this study. The observed direction of the changes of the ADP values caused by inclusion of relativistic correction (Table S5) is also in agreement with the previous study, while in the case of accounting for electron correlation, here we obtain the opposite result, namely, a decrease of the Au ADP values. The differences in the values of the corresponding diagonal *U*_11_, *U*_22_ and *U*_33_ components of the ADP tensor, similarly to our previous work, have an isotropic shape when REL and ECORR are taken into account (Tables S5 and S9), whereas when ANH and DIS are accounted for some anisotropy was observed (Tables S5 and S9). For the Au ADP, the amount of influence of the studied effects changes as follows: REL > ANH > ECORR >>> DIS ≈ SHADE, and for the carbon C25 the order is DIS >>> SHADE >> ECORR > ANH > REL.

### 3.3. Effect of modelling disorder on dynamic structure factors

**Figure 6:**
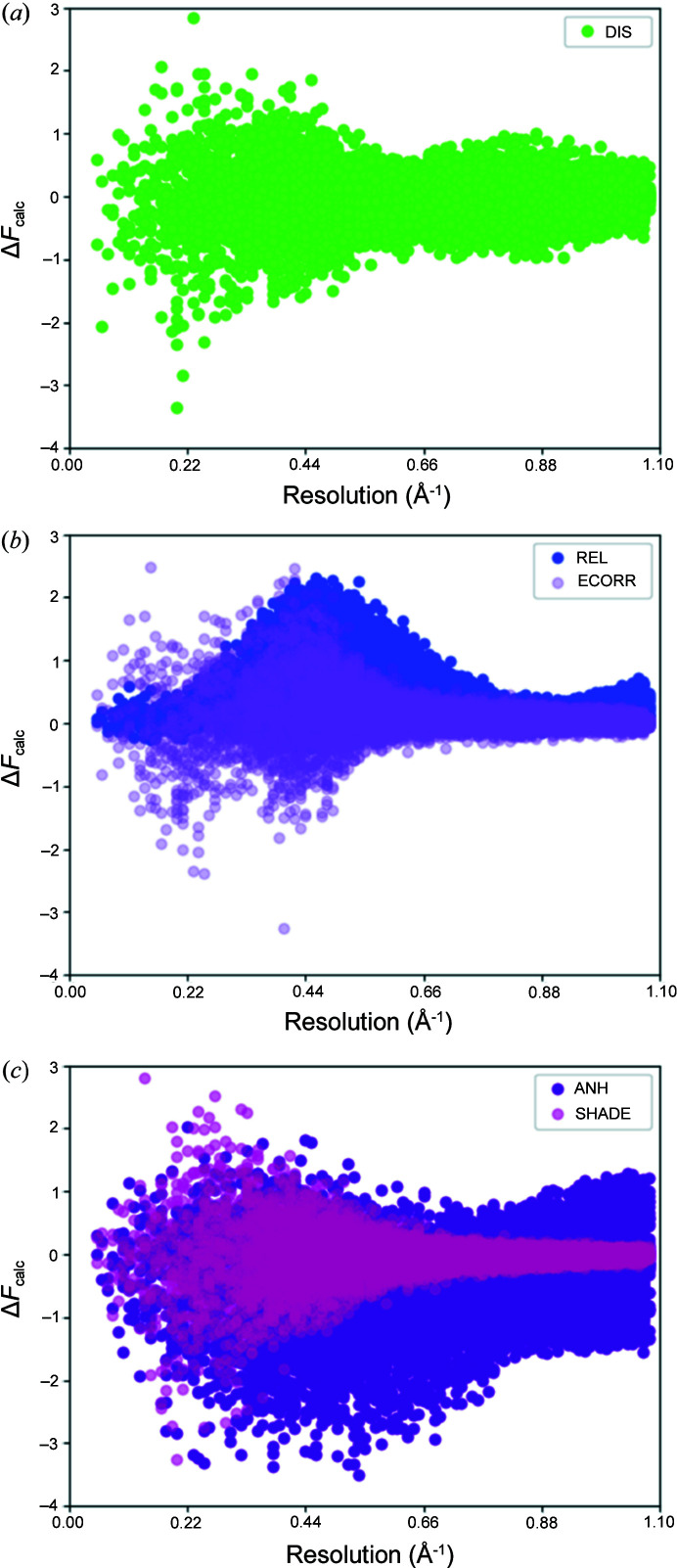
Differences in corresponding structure factors showing the effects of (*a*) disorder, (*b*) relativity and electron correlation, and (*c*) anharmonicity and SHADE. On the *x* axis is sinθ/λ (Å^−1^) up to the maximum experimental resolution. On the *y* axis is the difference of calculated structure factors in absolute scale (Δ*F*_calc_).

Here we inspect the amount of relativistic effects, electron correlation, disorder, anharmonicity and the effect of the treatment of hydrogen ADPs on the calculated dynamic structure factors. The differences of the calculated dynamic structure factors *versus* the data resolution (in Å^−1^) are shown in Fig. 6 ▸ in an absolute representation:

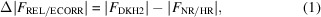




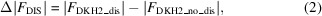




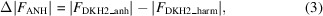




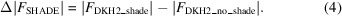

The electron correlation effect [Fig. 6 ▸(*b*)] is the most important for low-angle X-ray diffraction data (Bučinský *et al.*, 2016 ▸). It increases slightly with the data resolution and drops down approaching 0.70 A^−1^ after reaching the global maximum at around 0.45 A^−1^. On the other hand, structure factor differences due to relativistic effects [Fig. 6 ▸(*b*)] behave differently. For the resolution range used, two stepwise increases are observed. The most visible is in the range from the low-angle data, which shows little difference on inclusion of relativistic effects, up to the observed maximum at ∼0.50 A^−1^, followed by a decrease down to 0.80 A^−1^. In the higher-angle data region, starting from approximately 0.90 A^−1^ a slow re-increase is observed. These findings are in excellent agreement with the previously reported studies of resolution dependence of relativistic effects (Bučinský *et al.*, 2016 ▸; Fischer *et al.*, 2011 ▸; Batke & Eickerling, 2016*b* ▸,*a* ▸).

Among all effects visualized in Figs. 6 ▸(*a*), 6 ▸(*b*) and S2 of the supporting information, disorder has a slightly larger effect in the low-angle resolution region. However, similarly to electron correlation, some small increase is also observed, with the maximum around 0.40 A^−1^ resolution. When looking at the high-resolution data, refinement of disorder seems to influence structure factors the most in the region from 0.80 to 0.96 A^−1^. In Fig. 6 ▸(*c*) it can be seen that the low-angle data are also affected by treatment of hydrogen ADPs (SHADE). Similarly to the effects of electron correlation, SHADE influences low-angle X-ray data most, due to the fact that hydrogen atoms scatter at low sinθ/λ. The shape of the distribution of differences in the magnitudes of structure factors due to SHADE [Fig. 6 ▸(*c*)] is similar to the shape due to ECORR [Fig. 6 ▸(*b*)], although ECORR leans more towards positive *F* values, whereas SHADE is more or less symmetric around the *x* axis.

When inspecting consequences of accounting for anharmonicity of Au on the structure factors, two observations can be made immediately [Fig. 6 ▸(*c*)]. First, compared with all other investigated effects, anharmonicity takes the opposite course from low-angle data up to 0.60 A^−1^ and changes high-resolution structure factors the most. Second, the shape of the distribution complements the shape of the corresponding differences due to REL from low angles up to 0.80 Å^−1^, which is interesting as these two are the effects that impact Au directly.

**Figure 7:**
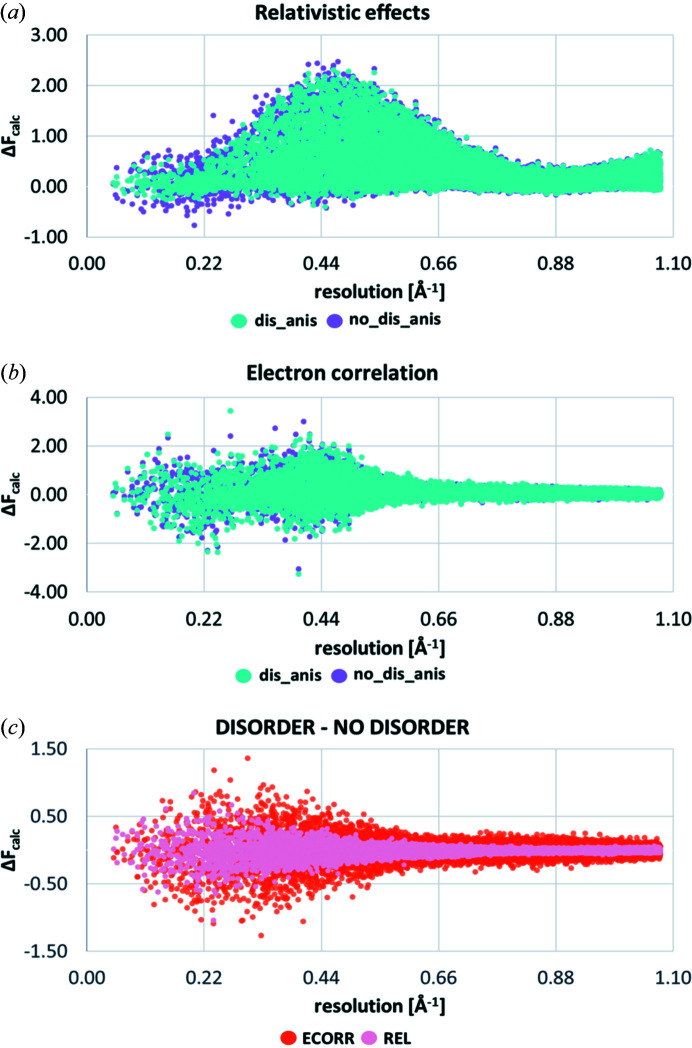
Differences in dynamic structure factors showing the effects of modelling disorder on the distribution of differences in the calculated dynamic structure factor amplitudes due to relativistic effects and electron correlation. On the *x* axis, sinθ/λ (Å^−1^) is plotted up to the maximum experimental resolution. On the *y* axis, the difference in calculated dynamic structure factors is plotted in absolute scale (Δ*F*_calc_).

In Fig. 7 ▸, we illustrate the effect of obtaining the quantum mechanical electron density from one molecular wavefunction or from merging two molecular wavefunctions according to the occupation factors of the two disorder components. When comparing these two models in the difference structure factors that represent electron correlation and relativistic effects, some small changes manifest in the low-angle regions [Figs. 7 ▸(*a*) and 7 ▸(*b*)]. A disorder model makes the distribution slightly narrower for REL in the resolution range from 0.06 to 0.45 Å^−1^ [Fig. 7 ▸(*a*)], whereas it makes it broader for ECORR [Fig. 7 ▸(*b*)]. Fig. 7 ▸(*c*) shows the regions of resolution of X-ray data in which the differences in structure factor moduli between REL_dis and REL_no_dis refinements as well as ECORR_dis and ECORR_no_dis occur. Both effects show similar global shapes, with points distributed fairly evenly along the *y* axis, whereas the differences are larger for ECORR compared with REL.

**Figure 8:**
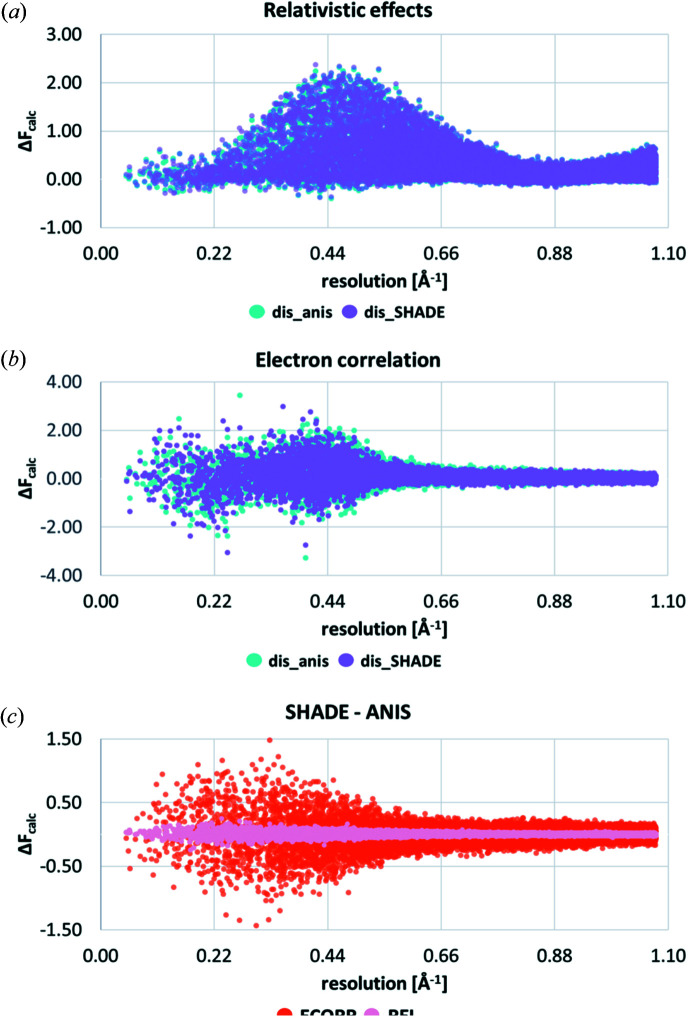
Differences in dynamic structure factors showing the effects of the treatment of hydrogen ADPs on the distribution of differences in the calculated dynamic structure factor amplitudes due to the relativistic effects and electron correlation. On the *x* axis, sinθ/λ (Å^−1^) is plotted up to the maximum experimental resolution. On the *y* axis, the difference in dynamic calculated structure factors is plotted in absolute scale (Δ*F*_calc_).

The same kind of comparison for the influence of hydrogen atom treatment on the dynamic structure factor differences is shown in Fig. 8 ▸. Figs. 8 ▸(*a*) and 8 ▸(*b*) present how accounting for relativistic effects and electron correlation changes the differences in the moduli of the dynamic structure factors when hydrogen ADPs were freely refined with HAR (anis) or estimated with the SHADE server (Madsen, 2006 ▸) and then constrained in HAR (in both cases the disorder was modelled). Overall, there is no significant change in the graph associated with the description of relativistic effects [Fig. 8 ▸(*a*)]. However, when looking at the distribution of differences in the corresponding structure factor moduli due to electron correlation, some fluctuations from low-angle data up to 0.55 A^−1^ are visible. This is depicted in Fig. 8 ▸(*b*). This effect is highlighted in Fig. 8 ▸(*c*) where the differences between SHADE and anis refinements are shown. Changes in the differences in the corresponding structure factor moduli for the case of relativistic effects are on the order of 0.1 × Δ*F*_calc_. For electron correlation, the changes are bigger and the description not notably different from the case of changes caused by accounting for disorder (Fig. 7 ▸). When inspecting the amplitude of differences in dynamic structure factors for REL, one obtains ∼2.4 and 1.7% of all structure factors affected by modelling disorder or treating hydrogen ADPs with SHADE, respectively. These values are slightly higher for electron correlation (5.3 and 7.7% of all structure factors for DIS and SHADE, respectively).

**Figure 9:**
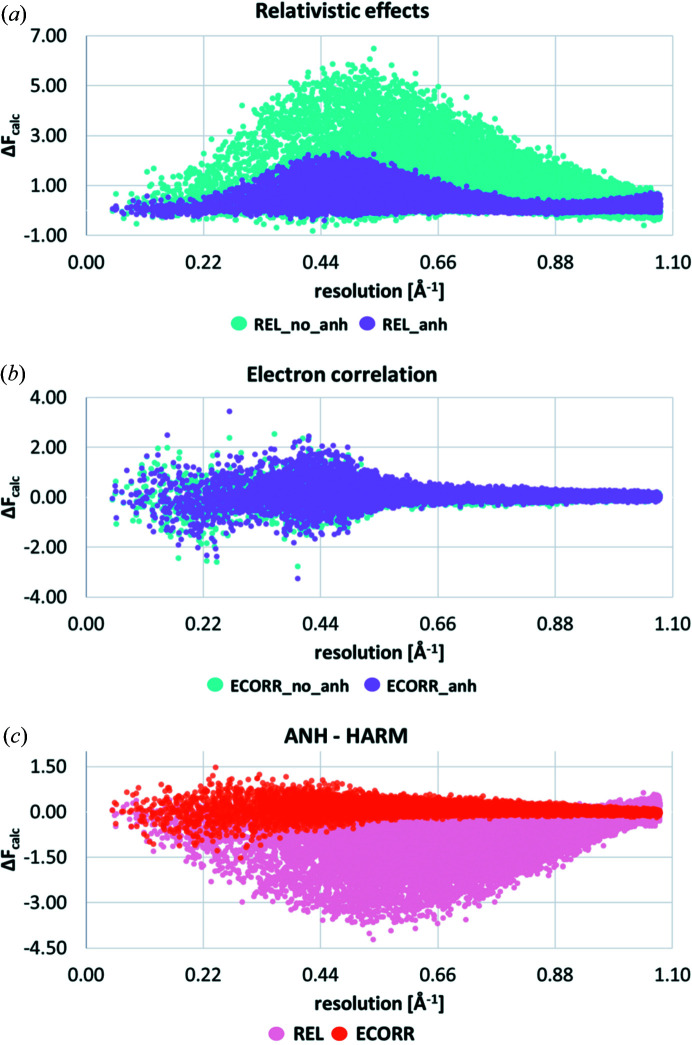
Differences in dynamic structure factors showing the effects of refinement of anharmonic thermal motion of Au ADP on the distribution of differences in the calculated dynamic structure factor amplitudes due to relativistic effects and electron correlation. On the *x* axis, sinθ/λ (Å^−1^) is plotted up to the maximum experimental resolution. On the *y* axis, the difference in calculated structure factors is plotted in absolute scale (Δ*F*_calc_).

The impact of the final investigated effect is shown in Fig. 9 ▸. The plots in Figs. 9 ▸(*a*) and 9 ▸(*b*) show the influence of the refinement of anharmonicity on the distributions of differences in calculated structure factors due to relativity and electron correlation. As expected, refinement of anharmonicity significantly changes the distribution of differences in calculated moduli of structure factors due to relativity over the whole range of examined resolutions as shown in Fig. 9 ▸(*a*). A key area is around 0.50 A^−1^, where a fourfold drop of the magnitude of Δ*F*_calc_ is observed on inclusion of anharmonicity. Generally, both the magnitude and the shape of the distribution change to more bell-shaped for the harmonic model and left-skewed for the anharmonic approach [Fig. 9 ▸(*a*)]. In contrast, the influence of the anharmonic motion on the difference dynamic structure factors affected by electron correlation is rather small, and mostly present for low-angle data, approximately up to 0.55 A^−1^ [Fig. 9 ▸(*b*)]. From Fig. 9 ▸(*c*) we can clearly see how disproportionate the effects of modelling anharmonic thermal motions for the cases of REL and ECORR are. In the case of accounting for the relativistic effects, almost 71% of all structure factors are affected by ANH, while for electron correlation only 7.8% of the structure factors changed.

## 4. Conclusions

A few important conclusions can be drawn from the results described above:

(1) Modelling disorder influences the dynamic structure factor moduli. These differences are differently pronounced in the signatures of relativistic and electron correlation effects in the structure factors. However, the changes in the case of ECORR are larger and mostly located in the region of the low-angle data.

(2) Treatment of hydrogen ADPs (refined *versus* fixed at SHADE values) only affects the distribution of differences in the calculated dynamic structure factor moduli due to electron correlation. Changes were observed from low-angle data up to 0.55 A^−1^, whereas in the case of relativistic effects they seemed to be irrelevant.

(3) Refinement of anharmonic thermal motion for Au has a significant impact on the distribution of differences in the calculated dynamic structure factor amplitudes due to relativistic effects, and is three times larger than for electron correlation.

(4) The signature of relativistic effects in the distribution of differences in structure factor amplitudes is strongly influenced by modelling the anharmonicity. It changes both the shape of the distribution and the moduli of the structure factors. On the other hand, disorder treatment only slightly affects the distribution of differences in structure factors, whereas SHADE has almost no influence on them.

(5) The shape of the distribution of differences in the moduli of the corresponding structure factors when electron correlation was considered and the amount of disorder modelled was very similar to the shape of the corresponding differences caused by the method of treating the hydrogen ADPs or the refinement of the anharmonic thermal motion for Au [Figs. 7 ▸(*b*), 8 ▸(*b*) and 9 ▸(*b*)], but the number of affected structure factors changes in the following order: SHADE ≈ ANH > DIS.

## 5. Summary and outlook

The focus of this work was to validate the modelling of disorder in HAR. A high-resolution and high-quality experimental X-ray diffraction dataset for the crystal structure of an organo-gold(I) compound was used. This successful benchmark study on modelling disorder for a compound with a heavy element was performed at different levels of theory, including HF and DFT with non-relativistic and DKH2 relativistic Hamiltonians. Within this study, we also explored the significance of modelling disorder on the results of HAR influenced separately by relativistic effects and electron correlation by analyzing changes of ADPs and differences in the corresponding moduli of dynamic structure factors. The role of disorder modelling was also compared with the effects of hydrogen ADP treatment and the refinement of atomic anharmonic thermal motions of Au.

Although the overall quality of the HAR models obtained did not change much when disorder was included (*R*-factors or maximum and minimum values of the residual density; Table 2 ▸), some small changes in the dynamic difference electron density maps were noticeable (Fig. 4 ▸). Of course, these changes are stronger near the atoms directly involved in the disorder. A more detailed analysis of Au and C25 ADPs showed that the disorder significantly changes carbon ADP values, but these changes are only marginal for Au. We also checked the influence of the other investigated effects on the Au and C25 ADPs. In the case of Au, it appears that the largest influences came from relativistic effects and atomic anharmonicity, whereas for carbon ADPs, the most significant are disorder and also the method employed to model hydrogen atoms. Electron correlation in both cases has a much smaller impact than the above-mentioned effects. We can also conclude that disorder and anharmonicity are quite independent of each other in this case, when looking at ADPs (Fig. 5 ▸ and Tables S5–12).

The differences between the investigated DIS, ANH and SHADE effects observed in the differences in moduli of the corresponding dynamic structure factors and their influence on the distribution of differences in the calculated dynamic structure factor amplitudes due to REL and ECORR were also compared. The distribution of the low-angle structure factors is mostly affected by the three effects: DIS, SHADE and ECORR (Fig. 6 ▸). Their shapes and magnitudes were found to be very similar, stressing the major influence of DIS and SHADE on the distribution of differences in the calculated dynamic structure factor amplitudes due to ECORR. On the other hand, distribution of differences in the calculated dynamic structure factor amplitudes due to ANH and REL tended to be very similar (Fig. 6 ▸). By contrast, DIS took the opposite course when looking at the distribution of differences of the calculated dynamic structure factors in the low-angle range (Fig. 6 ▸). Further inspection confirmed the importance of DIS, SHADE and ANH on the distribution of differences of the calculated dynamic structure factor amplitudes that account for electron correlation (Figs. 7 ▸–9 ▸ ▸). The changes in all structure factors is as follows: 7.8% > 7.7% > 5.3% for ANH, SHADE and DIS, respectively. For the relativistic effects, 71, 2.4 and 1.7% of all structure factors are affected when ANH, DIS and SHADE were included in the HAR refinements, respectively.

In our study, we could only indirectly assess the influence of the physical effects of electron correlation and the relativistic effects on dynamic structure factors and vice versa since the wavefunctions used for HAR are static, and the refined parameters only influence the wavefunctions via modified coordinates. In further studies an X-ray constrained wavefunction fitting procedure should be performed where the experimental structure factors directly modify the wavefunction parameters. However, this requires further software development to account for disorder and anharmonicity during the XCW fitting procedure. Only the effects of hydrogen ADP treatment on the XCW fitted wavefunctions could be investigated until now (Malaspina *et al.*, 2020 ▸).

The crystallographic data can be obtained freely via http://www.ccdc.cam.ac.uk/data_request/cif under CCDC deposition numbers 2159064–2159067, 2159374–2159377, 2160108, 2160109, 2160113, 2160115–2160120.

## 6. Related literature

The following reference is cited in the supporting information: Whitten & Spackman (2006 ▸).

## Supplementary Material

Crystal structure: contains datablock(s) IAM_disorder_dep. DOI: 10.1107/S2052252522005309/lt5049sup1.cif

Supporting information file. DOI: 10.1107/S2052252522005309/lt5049sup2.pdf

Zipped file containing cifs and check cifs. DOI: 10.1107/S2052252522005309/lt5049sup3.zip

CCDC reference: 2178113

## Figures and Tables

**Table:** 

Abbreviation	Method used
rks_nrel	DFT/PBE with non-relativistic Hamiltonian
rks_rel	DFT/PBE with relativistic DKH2 Hamiltonian
hf_rel	HF with relativistic DKH2 Hamiltonian
anis	Hydrogen ADPs obtained with HAR
shade	Hydrogen ADPs estimated with the SHADE3 server
dis	Model with disorder included
no_dis	Model with no disorder taken into account

**Table:** 

Abbreviation	Effect observed
REL	Relativistic effects
ECORR	Electron correlation
ANH	Anharmonicity
SHADE	Treatment of hydrogen atoms

**Table 2:** Comparison of agreement statistics for IAM and HAR models The HAR models presented here are obtained at the DFT/PBE level of theory with the DKH2 Hamiltonian and with hydrogen ADP values resulting from HAR (rks_rel_anis).

	No disorder	Disorder included
IAM		
Data/restraints/parameters	23743/0/337	23743/15/323†
GooF on *F* ^2^	1.091	1.092
Final *R* indexes (all data)	*R*_1_ = 2.16%, *w* *R*_2_ = 4.96%	*R*_1_ = 2.13%, *w* *R*_2_ = 4.89%
Largest difference peak/hole (eÅ^−3^)	2.07/−1.10	2.06/−1.11

HAR		
Data/restraints/parameters	23743/0/476	23743/30/524
GooF on *F* ^2^	0.993	0.990
Final *R* indexes (all data)	*R*_1_ = 1.81%, *w* *R*_2_ = 3.76%	*R*_1_ = 1.79%, *w* *R*_2_ = 3.73%
Largest difference peak/hole (eÅ)^−3^	1.01/−0.52	1.04/−0.51

† Due to the applied constraints, the number of parameters refined is smaller than for the ‘no disorder’ case
