# Supplementary material for: Influence of modelling disorder on Hirshfeld atom refinement results of an organo-gold(I) compound
Source: IUCrJ. 2022 Jun 11;9(Pt 4):497–507. doi: 10.1107/S2052252522005309 (PMC9252150; doi:10.1107/S2052252522005309)
Supplement: Supplementary file 2 [file m-09-00497-sup2.pdf]

IUCrJ

Volume 9 (2022)

Supporting information for article:

Influence of modelling disorder on Hirshfeld atom refinement results of an organo-gold(I) compound

Sylwia Pawlędzio, Maura Malinska, Florian Kleemiss, Simon Grabowsky and Krzysztof Woźniak

S1. Structural data – data processing and IAM refinement

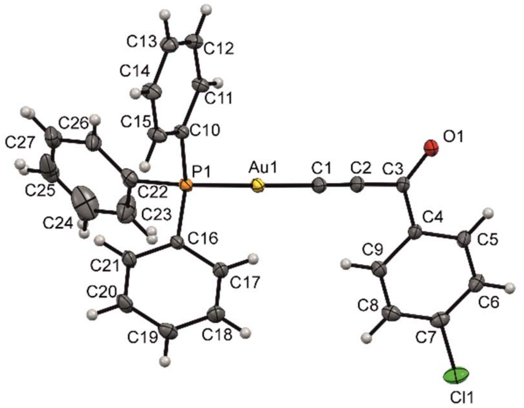

Figure S1 ORTEP-style representation of the molecule after IAM including disorder with labelling scheme. Ellipsoids are drawn at 50% probability level. H atoms are shown as a small spheres of arbitrary radius. The labelling scheme of disordered part has been omitted for the clarity.

Table S1 X-ray data collection and structure refinement details after IAM with disorder.

Radiation/Å	**SP8 (λ = 0.2482)**
Absorption correction	multi-scan
Empirical formula	$C_{27}H_{19}OPClAu$
Formula weight	622.81
Temperature/K	80
Crystal system	Monoclinic
Space group	C2/c
a/Å	17.7234(6)
b/Å	12.2442(5)
c/Å	21.3184(8)
α/°	90
β/°	94.6480(16)
γ/°	90
Volume/Å^3	4611.1(3)
Z	8
ρ_{calc}g/cm^3	1.794
μ/mm^{-1}	0.377
F(000)	2400.0
Crystal size/mm^3	0.079x0.025x0.021

2θ range for data collection/°	1.414 to 30.99
Index ranges	$-38 \le h \le 38$<br>$-26 \le k \le 26$<br>$-45 \le l \le 45$
Reflections collected	288963
Independent reflections	237430<br>R_{int} = 0.0582<br>R_{sigma} = 0.0221
Data/restraints/parameters	23743/15/323
Goodness-of-fit on F^2	1.092
Final R indexes [I>=2σ (I)]	R_1 = 0.0176, wR_2 = 0.0446
Final R indexes [all data]	R_1 = 0.0213, wR_2 = 0.0491
Largest diff. peak/hole / eÅ^{-3}	2.06/-1.11

S2. HAR refinements

Table S2 Statistical parameters of the all performed HARs.

	**H-atoms_anisotropic NO DISORDER**		
	**rks_nrel_anh**	**rhf_rel_anh**	**rks_rel_anh**
Data/restraints/parameters	23743/0/476	23738/0/476	23738/0/476
Goodness-of-fit on F^2	0.992	0.993	0.993
Final R indexes [I>=2σ (I)]	R_1 = 0.0145	R1 = 0.0144	R1 = 0.0146
	wR_2 = 0.0335	wR2 = 0.0333	wR2 = 0.0336
Final R indexes [all data]	R_1 = 0.0182	R1 = 0.0181	R1 = 0.0183
	wR_2 = 0.0379	wR2 = 0.0375	wR2 = 0.0380
Largest diff. peak/hole / eÅ^{-3}	1.04/-0.56	1.01/-0.53	1.05/-0.55
	**H-atoms_anisotropic DISORDER**		
	**rks_nrel_anh**	**rhf_rel_anh**	**rks_rel_anh**
Data/restraints/parameters	23743/30/524	23743/30/524	23743/30/524
Goodness-of-fit on F^2	0.991	0.989	0.990
Final R indexes [I>=2σ (I)]	R1 = 0.0141	R1 = 0.0141	R1 = 0.0142
	wR2 = 0.0327	wR2 = 0.0327	wR2 = 0.0328
Final R indexes [all data]	R_1 = 0.0178	R_1 = 0.0178	R_1 = 0.0179
	wR_2 = 0.0372	wR_2 = 0.0372	wR_2 = 0.0373
Largest diff. peak/hole / eÅ^{-3}	1.04/-0.55	1.04/-0.50	1.04/-0.51
	**H-atoms_anisotropic NO ANHARMONICITY**		
	**rks_nrel**	**rhf_rel**	**rks_rel**
Data/restraints/parameters	23743/30/499	23743/30/499	23743/30/499
Goodness-of-fit on F^2	0.996	1.006	1.003
Final R indexes [I>=2σ (I)]	R1 = 0.0155	R1 = 0.0158	R1 = 0.0157
	wR2 = 0.0358	wR2 = 0.0360	wR2 = 0.0358
Final R indexes [all data]	R_1 = 0.0193	R_1 = 0.0195	R_1 = 0.0195
	wR_2 = 0.0401	wR_2 = 0.0402	wR_2 = 0.0400

Largest diff. peak/hole / e$Å^{-3}$	2.33/-1.42	3.00/-1.08	3.02/-1.02

	**H-atoms_anisotropic NO ANHARMONICITY and NO DISORDER**		
	**rks_nrel**	**rhf_rel**	**rks_rel**
Data/restraints/parameters	23743/0/451	23738/0/451	23738/0/451
Goodness-of-fit on F^2	0.997	1.003	1.003
Final R indexes [I>=2σ (I)]	R1 = 0.0159<br>*w*R2 = 0.0366	R1 = 0.0160<br>*w*R2 = 0.0364	R1 = 0.0160<br>*w*R2 = 0.0362
Final R indexes [all data]	R_1 = 0.0197<br>wR_2 = 0.0408	R_1 = 0.0198<br>wR_2 = 0.0404	R_1 = 0.0197<br>wR_2 = 0.0403
Largest diff. peak/hole / e$Å^{-3}$	2.33/-1.43	3.04/-1.04	3.05/-0.98

	**H-atoms_SHADE NO DISORDER**		
	**rks_nrel_anh**	**rhf_rel_anh**	**rks_rel_anh**
Data/restraints/parameters	-	-	23743/0/362
Goodness-of-fit on F^2	-	-	0.992
Final R indexes [I>=2σ (I)]	-	-	R1 = 0.0147<br>*w*R2 = 0.0339
Final R indexes [all data]	-	-	R_1 = 0.0184<br>wR_2 = 0.0383
Largest diff. peak/hole / e$Å^{-3}$	-	-	1.04/-0.56

	**H-atoms_SHADE DISORDER**		
	**rks_nrel_anh**	**rhf_rel_anh**	**rks_rel_anh**
Data/restraints/parameters	23743/30/405	23743/30/405	23743/30/405
Goodness-of-fit on F^2	0.990	0.988	0.992
Final R indexes [I>=2σ (I)]	R1 = 0.0141<br>*w*R2 = 0.0328	R1 = 0.0143<br>*w*R2 = 0.0330	R1 = 0.0143<br>*w*R2 = 0.0329
Final R indexes [all data]	R_1 = 0.0178<br>wR_2 = 0.0373	R_1 = 0.0179<br>wR_2 = 0.0375	R_1 = 0.0179<br>wR_2 = 0.0374
Largest diff. peak/hole / e$Å^{-3}$	1.04/-0.52	1.04/-0.50	1.04/-0.51

S3. Atomic anharmonic thermal motions of Au

Table S3 3rd order of the Gram-Charlier coefficients obtained after HARs. Values higher than three standard uncertainties are highlighted in raspberry. The original values were multiplied by 10^7 for clarity.

	NO DISORDER H-atoms_anisotropic			DISORDER H-atoms_anisotropic			DISORDER H-atoms_SHADE		
	rks_nrel_anh	rhf_rel_anh	rks_rel_anh	rks_nrel_anh	rhf_rel_anh	rks_rel_anh	rks_nrel_anh	rhf_rel_anh	rks_rel_anh
U_{111}	-0.023(7)	-0.022(7)	-0.021(7)	-0.017(7)	-0.022(7)	-0.024(7)	-0.015(6)	-0.020(7)	-0.021(7)
U_{112}	0.164(6)	0.181(6)	0.177(6)	0.166(5)	0.184(6)	0.177(6)	0.166(5)	0.183(6)	0.178(6)
U_{113}	-0.021(3)	-0.023(3)	-0.023(3)	-0.02(3)	-0.023(3)	-0.022(3)	-0.019(3)	-0.023(3)	-0.023(3)
U_{122}	-0.28(9)	-0.29(9)	-0.288(9)	-0.275(9)	-0.291(9)	-0.291(9)	-0.274(9)	-0.291(9)	-0.289(9)
U_{123}	0.005(3)	0.004(3)	0.005(3)	0.005(3)	0.005(3)	0.005(3)	0.004(3)	0.005(3)	0.005(3)
U_{133}	-0.025(2)	-0.025(2)	-0.025(2)	-0.024(2)	-0.026(2)	-0.026(2)	-0.023(2)	-0.025(2)	-0.025(2)
U_{222}	0.89(3)	0.98(3)	0.96(3)	0.90(3)	1.01(3)	0.96(3)	0.91(3)	1.01(3)	0.97(3)
U_{223}	-0.154(7)	-0.159(7)	-0.16(7)	-0.152(7)	-0.162(7)	-0.159(7)	-0.152(7)	-0.161(7)	-0.161(7)
U_{233}	0.044(4)	0.052(4)	0.05(4)	0.045(4)	0.054(4)	0.049(4)	0.045(3)	0.053(4)	0.050(4)
U_{333}	-0.066(3)	-0.07(4)	-0.069(4)	-0.064(3)	-0.070(4)	-0.069(4)	-0.064(3)	-0.070(4)	-0.070(4)

Table S4 4th order of the Gram-Charlier coefficients obtained after HARs. Values higher than three standard uncertainties are highlighted in raspberry. The original values were multiplied by 10^8 for clarity.

	**NO DISORDER H-atoms_anisotropic**			**DISORDER H-atoms_anisotropic**			**DISORDER H-atoms_SHADE**		
	**rks_nrel_anh**	**rhf_rel_anh**	**rks_rel_anh**	**rks_nrel_anh**	**rhf_rel_anh**	**rks_rel_anh**	**rks_nrel_anh**	**rhf_rel_anh**	**rks_rel_anh**
U_{1111}	-0.021(3)	0.045(3)	0.038(3)	-0.020(3)	0.0046(3)	0.040(3)	-0.020(3)	0.044(3)	0.038(3)
U_{1112}	-0.0156(16)	-0.0183(17)	-0.0171(17)	-0.0152(16)	-0.00180(17)	-0.0170(17)	-0.0155(16)	-0.0180(17)	-0.0169(17)
U_{1113}	0.0042(10)	0.0085(10)	0.0085(10)	0.0044(9)	0.00084(10)	0.0086(10)	0.0041(9)	0.0082(10)	0.0085(10)
U_{1122}	-0.017(2)	0.031(2)	0.026(2)	-0.016(2)	0.0032(2)	0.027(2)	-0.017(2)	0.030(2)	0.026(2)
U_{1123}	-0.0037(7)	-0.0040(7)	-0.0040(8)	-0.0036(7)	-0.00040(7)	-0.0039(7)	-0.0036(7)	-0.0039(7)	-0.0039(7)
U_{1133}	-0.0063(6)	0.0087(6)	0.0072(6)	-0.0060(6)	0.00091(6)	0.0076(6)	-0.0062(6)	0.0086(6)	0.0073(6)
U_{1222}	-0.051(4)	-0.059(4)	-0.056(4)	-0.051(4)	-0.0058(4)	-0.056(4)	-0.051(4)	-0.058(4)	-0.055(4)
U_{1223}	0.0216(11)	0.0259(12)	0.0256(12)	0.0217(11)	0.00258(12)	0.0257(12)	0.0216(11)	0.0256(12)	0.0256(12)
U_{1233}	-0.0040(6)	-0.0043(6)	-0.0044(6)	-0.0041(6)	-0.00044(6)	-0.0044(6)	-0.0041(6)	-0.0043(6)	-0.0044(6)
U_{1333}	0.0037(6)	0.0068(7)	0.0069(7)	0.0037(6)	0.00068(6)	0.0069(6)	0.0037(6)	0.0066(7)	0.0068(6)
U_{2222}	-0.173(13)	0.139(15)	0.101(14)	-0.166(13)	0.146(14)	0.111(14)	-0.172(13)	0.138(14)	0.104(14)
U_{2223}	-0.030(3)	-0.035(3)	-0.033(3)	-0.030(3)	-0.035(3)	-0.033(3)	-0.030(3)	-0.035(3)	-0.033(3)
U_{2233}	-0.0186(13)	0.0136(14)	0.0103(14)	-0.0179(13)	0.0145(14)	0.0111(14)	-0.0184(13)	0.0132(14)	0.0105(14)
U_{2333}	-0.0079(8)	-0.0091(9)	-0.0086(9)	-0.0079(8)	-0.0090(9)	-0.0086(9)	-0.0078(8)	-0.0089(9)	-0.0086(9)
U_{3333}	-0.0106(11)	0.0199(13)	0.0169(13)	-0.0100(11)	0.0204(13)	0.0176(12)	-0.0104(11)	0.0192(13)	0.0171(12)

S4. Analysis of Au ADP obtained with different HAR models

Table S5 The ADP values (Å^2) obtained after different HARs with the three estimated standard deviations. The table presents also the difference between gold ADPs (Å^2) obtained from rks_nrel_anh_anis_dis, rhf_rel_anh_anis_dis and rks_rel_anh_anis_dis, rks_rel_anh_dis_shade HAR models including disorder. The REL_dis, ECORR_dis, ANH_dis, DIS and SHADE_dis representing the effects of relativity (rks_rel_anh_anis_dis – rks_nrel_anh_anis_dis), electron correlation (rks_rel_anh_anis_dis – rhf_rel_anh_anis_dis), anharmonicity (rks_rel_anh_anis_dis – rks_rel_no_anh_anis_dis), disorder (rks_rel_anh_anis_dis – rks_rel_anh_anis_no_dis) and treatment of hydrogen atom (rks_rel_anh_shade_dis – rks_rel_anh_anis_dis), respectively. Please note, that U_{ij} values for Au ADP obtained for rks_rel_anh_anis_no_dis and rks_rel_no_anh_anis_dis HAR model are present in Tables S6 and S8, respectively.

	rks nrel_anh_anis_dis	3esd	rhf rel_anh_anis_dis	3esd	rks rel_anh_anis_dis	3esd	rks rel_anh_dis_shade	3esd
U_{11}	1.59E-02	9.00E-05	1.71E-02	9.00E-05	1.696E-02	9.00E-05	1.69E-02	9.00E-05
U_{22}	2.10E-02	9.00E-05	2.22E-02	9.00E-05	2.205E-02	9.00E-05	2.20E-02	9.00E-05
U_{33}	1.23E-02	9.00E-05	1.34E-02	9.00E-05	1.330E-02	9.00E-05	1.33E-02	9.00E-05
U_{12}	-3.77E-03	4.50E-04	-3.80E-03	4.50E-04	-3.783E-03	4.50E-04	-3.78E-03	4.50E-05
U_{13}	1.98E-03	5.40E-04	2.06E-03	5.70E-04	2.065E-03	5.70E-04	2.06E-03	5.70E-05
U_{23}	-3.17E-03	4.20E-04	-3.19E-03	4.20E-04	-3.174E-03	4.20E-04	-3.17E-03	4.50E-05

REL_dis	ECORR_dis	ANH_dis	DIS	SHADE_dis
1.05E-03	-1.20E-04	5.05E-04	3.00E-05	-2.00E-05
1.10E-03	-1.40E-04	3.22E-04	3.00E-05	-2.00E-05
1.04E-03	-1.10E-04	4.46E-04	3.00E-05	-2.00E-05
-1.30E-05	1.30E-05	-2.17E-04	0.00E+00	1.00E-06
8.30E-05	9.00E-06	2.73E-04	0.00E+00	-2.00E-06
-9.00E-06	1.20E-05	-1.74E-04	0.00E+00	2.00E-06

Table S6 The ADP values (Å^2) obtained after different HARs with the three estimated standard deviations. The table presents also the difference between gold ADPs (Å^2) obtained from rks_nrel_anh_anis_no_dis, rhf_rel_anh_anis_no_dis and rks_rel_anh_anis_no_dis HAR models excluding disorder. The REL_no_dis, ECORR_no_dis and ANH_no_dis, representing the effects of relativity (rks_rel_anh_anis_no_dis – rks_nrel_anh_anis_no_dis), electron correlation (rks_rel _anh_anis_no_dis – rhf_rel_anh_anis_no_dis) and anharmonicity (rks_rel_anh_anis_no_dis – rks_rel_no_anh_anis_no_dis), respectively. Please note, that U_{ij} values for Au ADP obtained for rks_rel_no_anh_anis_no_dis HAR model are present in Table S7.

	rks nrel_anh_anis_no_dis	3esd	rhf rel_anh_anis_no_dis	3esd	rks rel_anh_anis_no_dis	3esd
U_{11}	1.59E-02	9.00E-05	1.71E-02	9.00E-05	1.693E-02	9.00E-05
U_{22}	2.09E-02	9.00E-05	2.22E-02	9.00E-05	2.202E-02	9.00E-05
U_{33}	1.22E-02	9.00E-05	1.34E-02	9.00E-05	1.327E-02	9.00E-05
U_{12}	-3.77E-03	4.50E-04	-3.80E-03	4.50E-04	-3.783E-03	4.80E-04
U_{13}	1.98E-03	5.40E-04	2.06E-03	5.70E-04	2.065E-03	5.70E-04
U_{23}	-3.17E-03	4.20E-04	-3.19E-03	4.50E-04	-3.174E-03	4.50E-04

REL_no_dis	ECORR_no_dis	ANH_no_dis
1.04E-03	-1.30E-04	4.76E-04
1.09E-03	-1.50E-04	2.93E-04
1.03E-03	-1.20E-04	4.20E-04
-1.20E-05	1.50E-05	-2.17E-04
8.20E-05	5.00E-06	2.72E-04
-9.00E-06	1.30E-05	-1.75E-04

Table S7 The ADP values (Å^2) obtained after different HARs with the three estimated standard deviations. The table presents also the difference between gold ADPs (Å^2) obtained from rks_nrel_no_anh_anis_no_dis, rhf_rel_no_anh_anis_no_dis and rks_rel_no_anh_anis_no_dis HAR models excluding disorder and atomic anharmonic thermal motions of Au. The REL_ no_anh_no_dis and ECORR_no_anh_no_dis representing the effects of relativity (rks_rel_no_anh_anis_no_dis – rks_nrel_no_anh_anis_no_dis) and electron correlation (rks_rel_no_anh_anis_no_dis – rhf_rel_no_anh_anis_no_dis), respectively.

	rks nrel_no_anh_anis_no_dis	3esd	rhf rel_no_anh_anis_no_dis	3esd	rks rel_no_anh_anis_no_dis	3esd	REL no_anh_no_dis	ECORR no_anh_no_dis
U_{11}	1.62E-02	3.00E-05	1.65E-02	2.70E-05	1.65E-02	2.70E-05	2.42E-04	-3.50E-05
U_{22}	2.14E-02	3.30E-05	2.18E-02	3.30E-05	2.17E-02	3.30E-05	2.80E-04	-4.20E-05
U_{33}	1.26E-02	2.70E-05	1.29E-02	2.70E-05	1.29E-02	2.70E-04	2.29E-04	-3.10E-05
U_{12}	-3.56E-03	1.80E-05	-3.57E-03	1.80E-05	-3.566E-03	1.80E-05	-8.00E-06	5.00E-06
U_{13}	1.78E-03	1.80E-05	1.79E-03	1.80E-05	1.793E-03	1.80E-05	1.60E-05	1.00E-06
U_{23}	-2.99E-03	1.80E-05	-3.00E-03	1.80E-05	-2.999E-03	1.80E-05	-7.00E-06	5.00E-06

Table S8 The ADP values (Å^2) obtained after different HARs with the three estimated standard deviations. The table presents also the difference between gold ADPs (Å^2) obtained from rks_nrel_no_anh_anis_dis, rhf_rel_no_anh_anis_dis and rks_rel_no_anh_anis_dis HAR models including disorder and excluding atomic anharmonic thermal motions of Au. The REL_ no_anh and ECORR_ no_anh represent the effects of relativity (rks_rel_no_anh_anis_dis – rks_nrel_no_anh_anis_dis) and electron correlation (rks_rel_no_anh_anis_dis – rhf_rel_no_anh_anis_dis), respectively.

	rks nrel_no_anh_anis_dis	3esd	rhf rel_no_anh_anis_dis	3esd	rks rel_no_anh_anis_dis	3esd	REL no_anh	ECORR no_anh
U_{11}	1.62E-02	2.70E-05	1.65E-02	2.70E-05	1.646E-02	2.70E-05	2.40E-04	-3.40E-05
U_{22}	2.15E-02	3.30E-05	2.18E-02	3.30E-05	2.173E-02	3.30E-05	2.77E-04	-4.10E-05
U_{33}	1.26E-02	2.70E-04	1.29E-02	2.70E-05	1.285E-02	2.70E-05	2.30E-04	-3.00E-05
U_{12}	-3.56E-03	1.80E-05	-3.57E-03	1.80E-05	-3.566E-03	1.80E-05	-7.00E-06	5.00E-06
U_{13}	1.78E-03	1.80E-05	1.79E-03	1.80E-05	1.792E-03	1.80E-05	1.60E-05	2.00E-06
U_{23}	-2.99E-03	1.80E-05	-3.01E-03	1.80E-05	-3.000E-03	1.80E-05	-7.00E-06	5.00E-06

S5. Analysis of C25 ADP obtained with different HAR models

Table S9 The ADP values (Å^2) obtained after different HARs with the three estimated standard deviations. The table presents also the difference between C25 ADPs (Å^2) obtained from rks_nrel_anh_anis_dis, rhf_rel_anh_anis_dis and rks_rel_anh_anis_dis, rks_rel_anh_dis_shade HAR models including disorder. The REL_dis, ECORR_dis, ANH_dis, DIS and SHADE_dis representing the effects of relativity (rks_rel_anh_anis_dis – rks_nrel_anh_anis_dis), electron correlation (rks_rel_anh_anis_dis – rhf_rel_anh_anis_dis), anharmonicity (rks_rel_anh_anis_dis – rks_rel_no_anh_anis_dis), disorder (rks_rel_anh_anis_dis – rks_rel_anh_anis_no_dis) and treatment of hydrogen atom (rks_rel_anh_shade_dis – rks_rel_anh_anis_dis), respectively. Please note, that U_{ij} values for C25 ADP obtained for rks_rel_anh_anis_no_dis and rks_rel_no_anh_anis_dis HAR model are present in Tables S10 and S12, respectively.

	rks nrel_anh_anis_dis	3esd	rhf rel_anh_anis_dis	3esd	rks rel_anh_anis_dis	3esd	rks rel_anh_dis_shade	3esd
U_{11}	3.36E-02	2.10E-03	0.0327	2.10E-03	3.34E-02	2.10E-03	3.17E-02	2.40E-03
U_{22}	1.99E-02	1.20E-03	2.02E-02	1.20E-03	1.98E-02	1.20E-03	2.00E-02	1.50E-03
U_{33}	2.23E-02	1.50E-03	2.29E-02	1.50E-03	2.22E-02	1.50E-03	2.25E-02	1.80E-03
U_{12}	4.40E-03	9.00E-04	4.40E-03	9.00E-04	4.40E-03	9.00E-04	4.10E-03	9.00E-04
U_{13}	-9.00E-04	1.20E-03	7.00E-04	1.20E-03	-9.00E-04	1.20E-03	-1.00E-03	1.20E-03
U_{23}	-2.00E-04	9.00E-04	3.00E-04	9.00E-04	-2.00E-04	9.00E-04	-4.00E-04	1.20E-03

REL_dis	ECORR_dis	ANH_dis	DIS	SHADE
-2.00E-04	7.00E-04	-1.00E-04	-8.20E-03	1.70E-03
-1.00E-04	-4.00E-04	-3.00E-04	-8.00E-04	-2.00E-04
-1.00E-04	-7.00E-04	-3.00E-04	-8.00E-04	-3.00E-04
0.00E+00	0.00E+00	-2.00E-04	-5.00E-04	3.00E-04
0.00E+00	-1.60E-03	-2.00E-04	-1.10E-03	1.00E-04
0.00E+00	-5.00E-04	1.00E-04	0.00E+00	2.00E-04

Table S10 The ADP values (Å^2) obtained after different HARs with the three estimated standard deviations. The table presents also the difference between C25 ADPs (Å^2) obtained from rks_nrel_anh_anis_no_dis, rhf_rel_anh_anis_no_dis and rks_rel_anh_anis_no_dis HAR models excluding disorder. The REL_no_dis, ECORR_no_dis and ANH_no_dis, representing the effects of relativity (rks_rel_anh_anis_no_dis – rks_nrel_anh_anis_no_dis), electron correlation (rks_rel _anh_anis_no_dis – rhf_rel_anh_anis_no_dis) and anharmonicity (rks_rel_anh_anis_no_dis – rks_rel_no_anh_anis_no_dis), respectively. Please note, that U_{ij} values for C25 ADP obtained for rks_rel_no_anh_anis_no_dis HAR model are present in Table S11.

	rks nrel_anh_anis_no_dis	3esd	rhf rel_anh_anis_no_dis	3esd	rks rel_anh_anis_no_dis	3esd
U_{11}	4.18E-02	1.20E-03	4.12E-02	1.20E-03	4.16E-02	1.20E-03
U_{22}	2.07E-02	9.00E-04	2.06E-02	9.00E-04	2.06E-02	9.00E-04
U_{33}	2.31E-02	9.00E-04	2.32E-02	9.00E-04	2.30E-02	9.00E-04
U_{12}	5.00E-03	9.00E-03	4.80E-03	9.00E-04	4.90E-03	9.00E-04
U_{13}	2.00E-04	9.00E-04	3.00E-04	9.00E-04	2.00E-04	9.00E-04
U_{23}	-2.00E-04	6.00E-04	-2.00E-04	6.00E-04	-2.00E-04	6.00E-04

REL_no_dis	ECORR_no_dis	ANH_no_dis
-2.00E-04	4.00E-04	3.00E-04
-1.00E-04	0.00E+00	1.00E-04
-1.00E-04	-2.00E-04	1.00E-04
-1.00E-04	1.00E-04	0.00E+00
0.00E+00	-1.00E-04	0.00E+00
0.00E+00	0.00E+00	0.00E+00

Table S11 The ADP values (Å^2) obtained after different HARs with the three estimated standard deviations. The table presents also the difference between gold ADPs (Å^2) obtained from rks_nrel_no_anh_anis_no_dis, rhf_rel_no_anh_anis_no_dis and rks_rel_no_anh_anis_no_dis HAR models excluding disorder and atomic anharmonic thermal motions of Au. The REL_no_anh_no_dis and ECORR_no_anh_no_dis representing the effects of relativity (rks_rel_no_anh_anis_no_dis – rks_nrel_no_anh_anis_no_dis) and electron correlation (rks_rel_no_anh_anis_no_dis – rhf_rel_no_anh_anis_no_dis), respectively.

	rks nrel_no_anh_anis_no_dis	3esd	rhf rel_no_anh_anis_no_dis	3esd	rks rel_no_anh_anis_no_dis	3esd	REL no_anh_no_dis	ECORR no_anh_no_dis
U_{11}	4.19E-02	1.50E-03	0.041	1.20E-03	4.13E-02	1.20E-03	-6.00E-04	3.00E-04
U_{22}	2.10E-02	9.00E-04	0.0205	9.00E-04	2.05E-02	9.00E-04	-5.00E-04	0.00E+00
U_{33}	2.33E-02	9.00E-04	2.31E-02	9.00E-04	2.29E-02	9.00E-04	-4.00E-04	-2.00E-04
U_{12}	4.90E-03	9.00E-04	4.80E-03	9.00E-04	4.90E-03	9.00E-04	0.00E+00	1.00E-04
U_{13}	2.00E-04	9.00E-04	3.00E-04	9.00E-04	2.00E-04	9.00E-04	0.00E+00	-1.00E-04
U_{23}	-2.00E-04	6.00E-04	-1.00E-04	6.00E-04	-2.00E-04	6.00E-04	0.00E+00	-1.00E-04

Table S12 The ADP values (Å²) obtained after different HARs with the three estimated standard deviations. The table presents also the difference between C25 ADPs (Å²) obtained from rks_nrel_no_anh_anis_dis, rhf_rel_no_anh_anis_dis and rks_rel_no_anh_anis_dis HAR models including disorder and excluding atomic anharmonic thermal motions of Au. The REL_ no_anh and ECORR_ no_anh represent the effects of relativity (rks_rel_no_anh_anis_dis – rks_nrel_no_anh_anis_dis) and electron correlation (rks_rel_no_anh_anis_dis – rhf_rel_no_anh_anis_dis), respectively.

	rks nrel_no_anh_anis_dis	3esd	rhf rel_no_anh_anis_dis	3esd	rks rel_no_anh_anis_dis	3esd	REL no_anh	ECORR no_anh
U_{11}	3.22E-02	3.00E-03	3.23E-02	2.40E-03	3.35E-02	2.10E-03	1.30E-03	1.20E-03
U_{22}	2.03E-02	1.80E-03	2.01E-02	1.50E-03	2.01E-02	1.20E-03	-2.00E-04	0.00E+00
U_{33}	2.20E-02	2.10E-03	2.29E-02	1.50E-03	2.25E-02	1.20E-03	5.00E-04	-4.00E-04
U_{12}	4.20E-03	1.20E-03	4.40E-03	1.20E-03	4.60E-03	9.00E-04	4.00E-04	2.00E-04
U_{13}	-1.00E-03	1.50E-03	-7.00E-04	1.50E-03	-7.00E-04	1.20E-03	3.00E-04	0.00E+00
U_{23}	-8.00E-04	1.20E-03	-4.00E-04	1.20E-03	-3.00E-04	9.00E-04	5.00E-04	1.00E-04

S6. Similarity index

The comparison of the ADPs obtained from HAR and estimated with SHADE3 has been performed by using similarity index S_{12} (S_{12}= 1000(1 – R_{12})) which describes a percentage between two probability density functions (pdfs) expressed as ADPs. In above equation, R_{12} describes the overlap between the p1 and p2 pdfs of the analysed ADPs tensors[1] and is defined as:

$$R_{12} = \int [p_1(x)p_2(x)]^{1/2} d^3x = \frac{2^{3/2}(det U_1^{-1} U_2^{-1})^{1/4}}{[\det(U_1^{-1} + U_2^{-1})]^{1/2}}$$

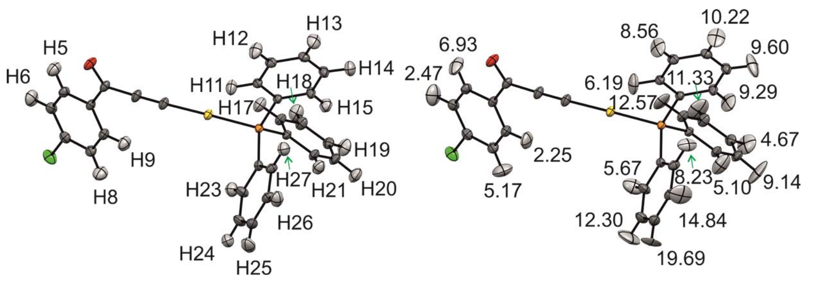

Figure S2 Similarity index (S12) calculated for the hydrogen atom ADPs obtained from rks_rel_anh_shade_dis (left) and rks_rel_anh_anis_dis (right). In both cases, major component of the disorder was taken. S12 indices are given for each H atom.

As one can see, the obtained S_{12} values ranged from ~2.5 to 20 %. In all cases, the H atom ADPs obtained from HAR are elongated in some directions, when compared to those estimated by SHADE.

References:

Whitten, A.E.; Spackman, M.A. Anisotropic Displacement Parameters for H Atoms Using an ONIOM Approach. Acta Crystallogr. B 2006, 62, 875–888.
