# Supplementary material for: Influence of modelling disorder on Hirshfeld atom refinement results of an organo-gold(I) compound
Source: IUCrJ. 2022 Jun 11;9(Pt 4):497–507. doi: 10.1107/S2052252522005309 (PMC9252150; doi:10.1107/S2052252522005309)

checkCIF/PLATON report

You have not supplied any structure factors. As a result the full set of tests cannot be run.

THIS REPORT IS FOR GUIDANCE ONLY. IF USED AS PART OF A REVIEW PROCEDURE FOR PUBLICATION, IT SHOULD NOT REPLACE THE EXPERTISE OF AN EXPERIENCED CRYSTALLOGRAPHIC REFEREE.

No syntax errors found. CIF dictionary Interpreting this report

Datablock: mm_apex3

```
Bond precision:   C-C = 0.0012 A           Wavelength=0.24820

Cell:             a=17.7234(6)     b=12.2442(5)         c=21.3184(8)
                  alpha=90         beta=94.6480(16)     gamma=90
Temperature:      80 K

                 Calculated                   Reported
Volume           4611.1(3)                    4611.1(3)
Space group      C 2/c                        C 1 2/c 1
Hall group       -C 2yc                       -C 2yc
Moiety formula   C27 H19 Au Cl O P            C27 H19 Au Cl O P
Sum formula      C27 H19 Au Cl O P            C27 H19 Au Cl O P
Mr               622.81                       622.81
Dx,g cm-3        1.794                        1.794
Z                8                            8
Mu (mm-1)        0.439                        0.377
F000             2400.0                       2400.0
F000'            2390.61
h,k,lmax         38,26,45                     38,26,45
Nref             24077                        23743
Tmin,Tmax        0.967,0.978                  0.663,0.744
Tmin'            0.967

Correction method= # Reported T Limits: Tmin=0.663 Tmax=0.744
AbsCorr = MULTI-SCAN

Data completeness= 0.986            Theta(max)= 15.495

R(reflections)= 0.0176( 21044)                         wR2(reflections)=
                                                       0.0491( 23743)
S = 1.092                    Npar= 323
```

The following ALERTS were generated. Each ALERT has the format
test-name_ALERT_alert-type_alert-level.
Click on the hyperlinks for more details of the test.

Alert level A

```
PLAT051_ALERT_1_A Mu(calc) and Mu(CIF) Ratio Differs from 1.0 by .      16.52 %
```

Author Response: Here, we are dealing with wavelengths from the synchrotron an thus the calculation of _exptl_absorpt_correction_mu should be skipped.

Alert level C

```
PLAT220_ALERT_2_C NonSolvent   Resd 1  C   Ueq(max)/Ueq(min) Range        3.3 Ratio
```

Author Response: This alert is generated because there is a disorder in the structure.

Alert level G

```
ABSMU01_ALERT_1_G  Calculation of _exptl_absorpt_correction_mu
                not performed for this radiation type.
PLAT002_ALERT_2_G Number of Distance or Angle Restraints on AtSite         13 Note
PLAT012_ALERT_1_G N.O.K.   _shelx_res_checksum Found in CIF ......     Please Check
PLAT019_ALERT_1_G _diffrn_measured_fraction_theta_full/*_max < 1.0      0.986 Report
PLAT092_ALERT_4_G Check: Wavelength Given is not Cu,Ga,Mo,Ag,In Ka    0.24820 Ang.
PLAT176_ALERT_4_G The CIF-Embedded .res File Contains SADI Records         15 Report
PLAT232_ALERT_2_G Hirshfeld Test Diff (M-X)  Au1      --C1       .        7.7 s.u.
PLAT301_ALERT_3_G Main Residue  Disorder .............(Resd  1  )         19% Note
PLAT371_ALERT_2_G Long   C(sp2)-C(sp1) Bond  C2       - C3       .       1.44 Ang.
PLAT434_ALERT_2_G Short Inter HL..HL Contact Cl1      ..Cl1      .       3.25 Ang.
                                                -x,-y,1-z  =      5_556 Check
PLAT860_ALERT_3_G Number of Least-Squares Restraints ............          15 Note
PLAT933_ALERT_2_G Number of HKL-OMIT Records in Embedded .res File         31 Note
PLAT984_ALERT_1_G The Au-f'=   -1.0970 Deviates from the B&C-Value    -1.0922 Check
PLAT985_ALERT_1_G The Au-f"=    1.5108 Deviates from the B&C-Value     1.5133 Check
```

---

1 **ALERT level A** = Most likely a serious problem - resolve or explain
0 **ALERT level B** = A potentially serious problem, consider carefully
1 **ALERT level C** = Check. Ensure it is not caused by an omission or oversight
14 **ALERT level G** = General information/check it is not something unexpected

6 ALERT type 1 CIF construction/syntax error, inconsistent or missing data
6 ALERT type 2 Indicator that the structure model may be wrong or deficient
2 ALERT type 3 Indicator that the structure quality may be low
2 ALERT type 4 Improvement, methodology, query or suggestion
0 ALERT type 5 Informative message, check

It is advisable to attempt to resolve as many as possible of the alerts in all categories. Often the minor alerts point to easily fixed oversights, errors and omissions in your CIF or refinement strategy, so attention to these fine details can be worthwhile. In order to resolve some of the more serious problems it may be necessary to carry out additional measurements or structure refinements. However, the purpose of your study may justify the reported deviations and the more serious of these should normally be commented upon in the discussion or experimental section of a paper or in the "special_details" fields of the CIF. checkCIF was carefully designed to identify outliers and unusual parameters, but every test has its limitations and alerts that are not important in a particular case may appear. Conversely, the absence of alerts does not guarantee there are no aspects of the results needing attention. It is up to the individual to critically assess their own results and, if necessary, seek expert advice.

Publication of your CIF in IUCr journals

A basic structural check has been run on your CIF. These basic checks will be run on all CIFs submitted for publication in IUCr journals (*Acta Crystallographica*, *Journal of Applied Crystallography*, *Journal of Synchrotron Radiation*); however, if you intend to submit to *Acta Crystallographica Section C* or *E* or *IUCrData*, you should make sure that full publication checks are run on the final version of your CIF prior to submission.

Publication of your CIF in other journals

Please refer to the *Notes for Authors* of the relevant journal for any special instructions relating to CIF submission.

PLATON version of 20/01/2022; check.def file version of 19/01/2022

Datablock mm_apex3 - ellipsoid plot

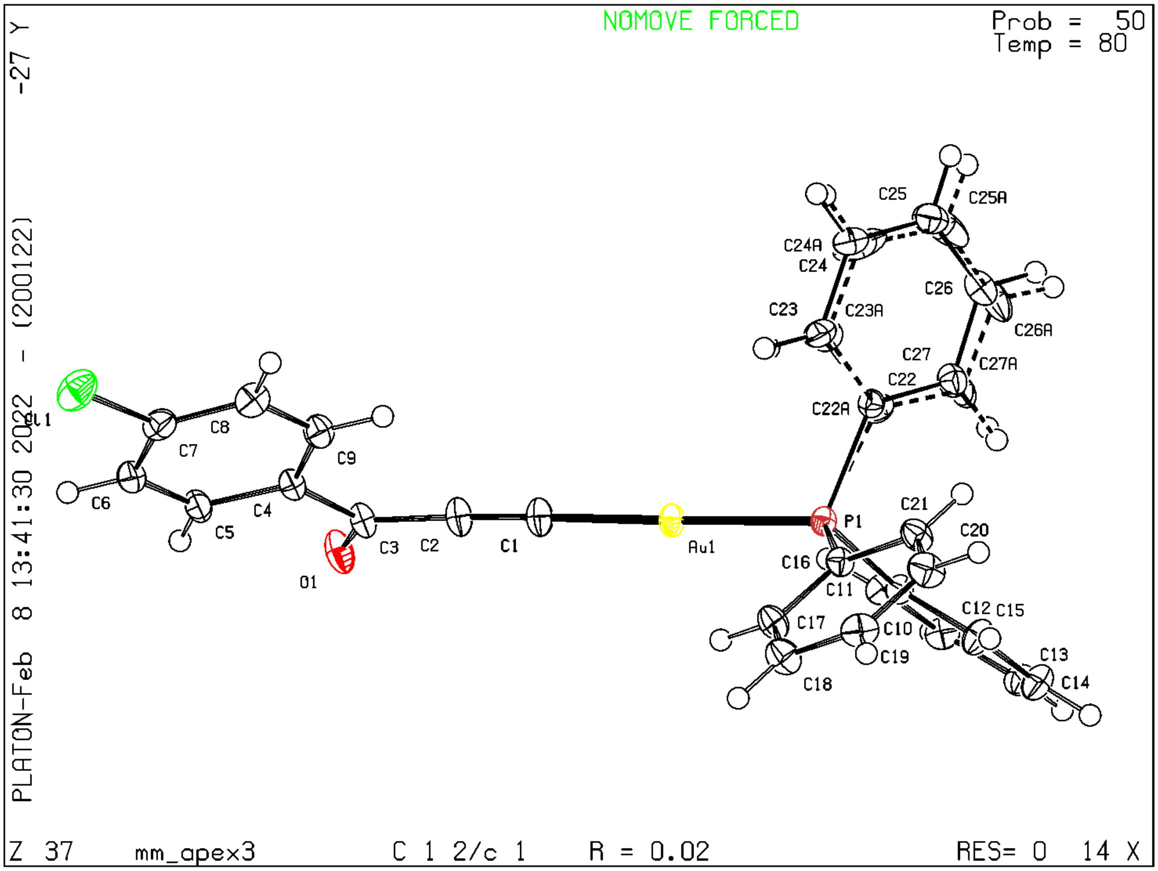

Supplement: Supplementary file 3 [file m-09-00497-sup3.zip › CIFs_with_check_cif/IAM_disorder-dep_checkcif.pdf]
