# Supplementary material for: Influence of modelling disorder on Hirshfeld atom refinement results of an organo-gold(I) compound
Source: IUCrJ. 2022 Jun 11;9(Pt 4):497–507. doi: 10.1107/S2052252522005309 (PMC9252150; doi:10.1107/S2052252522005309)

checkCIF/PLATON report

Structure factors have been supplied for datablock(s) mm_apex3

THIS REPORT IS FOR GUIDANCE ONLY. IF USED AS PART OF A REVIEW PROCEDURE FOR PUBLICATION, IT SHOULD NOT REPLACE THE EXPERTISE OF AN EXPERIENCED CRYSTALLOGRAPHIC REFEREE.

No syntax errors found. CIF dictionary Interpreting this report

Datablock: mm_apex3

Bond precision: C-C = 0.0010 A Wavelength=0.24820

Cell:	a=17.7234(6)	b=12.2442(5)	c=21.3184(8)
	alpha=90	beta=94.6480(16)	gamma=90
Temperature:	80 K		

	Calculated	Reported
Volume	4611.1(3)	4611.1(3)
Space group	C 2/c	C 1 2/c 1
Hall group	-C 2yc	-C 2yc
Moiety formula	C27 H19 Au Cl O P	C27 H19 Au Cl O P
Sum formula	C27 H19 Au Cl O P	C27 H19 Au Cl O P
Mr	622.81	622.84
Dx,g cm-3	1.794	1.794
Z	8	8
Mu (mm-1)	0.439	0.377
F000	2400.0	2390.6
F000’	2390.61	
h,k,lmax	38,26,45	38,26,45
Nref	24056	23743
Tmin,Tmax	0.967,0.978	0.663,0.744
Tmin’	0.967	

Correction method= # Reported T Limits: Tmin=0.663 Tmax=0.744
AbsCorr = MULTI-SCAN

Data completeness= 0.987 Theta(max)= 15.490

R(reflections)= 0.0147(21044) wR2(reflections)= 0.0383(23743)

S = 0.992 Npar= 362

The following ALERTS were generated. Each ALERT has the format
test-name_ALERT_alert-type_alert-level.
Click on the hyperlinks for more details of the test.

Alert level A

PLAT051_ALERT_1_A Mu(calc) and Mu(CIF) Ratio Differs from 1.0 by . 16.52 %

Author Response: Here, we are dealing with wavelengths from the synchrotron an thus the calculation of _exptl_absorpt_correction_mu should be skipped.

Alert level B

PLAT934_ALERT_3_B Number of (Iobs-Icalc)/Sigma(W) > 10 Outliers .. 7 Check

Author Response: This is a consequence of the presence of disorder.

Alert level C

PLAT218_ALERT_3_C Constrained U(ij) Components(s) for H9 . 6 Check

Author Response: The ADP values for hydrogen atoms were estimated using SHADE3.

PLAT218_ALERT_3_C Constrained U(ij) Components(s) for H15 . 6 Check

Author Response: The ADP values for hydrogen atoms were estimated using SHADE3.

PLAT218_ALERT_3_C Constrained U(ij) Components(s) for H11 . 6 Check

Author Response: The ADP values for hydrogen atoms were estimated using SHADE3.

PLAT218_ALERT_3_C Constrained U(ij) Components(s) for H5 . 6 Check

Author Response: The ADP values for hydrogen atoms were estimated using SHADE3.

PLAT218_ALERT_3_C Constrained U(ij) Components(s) for H19 . 6 Check

Author Response: The ADP values for hydrogen atoms were estimated using SHADE3.

PLAT218_ALERT_3_C Constrained U(ij) Components(s) for H27 . 6 Check

Author Response: The ADP values for hydrogen atoms were estimated using SHADE3.

PLAT218_ALERT_3_C Constrained U(ij) Components(s) for H21 . 6 Check

Author Response: The ADP values for hydrogen atoms were estimated using SHADE3.

PLAT218_ALERT_3_C Constrained U(ij) Components(s) for H25 . 6 Check

Author Response: The ADP values for hydrogen atoms were estimated using SHADE3.

PLAT218_ALERT_3_C Constrained U(ij) Components(s) for H26 . 6 Check

Author Response: The ADP values for hydrogen atoms were estimated using SHADE3.

PLAT218_ALERT_3_C Constrained U(ij) Components(s) for H6 . 6 Check

Author Response: The ADP values for hydrogen atoms were estimated using SHADE3.

PLAT218_ALERT_3_C Constrained U(ij) Components(s) for H12 . 6 Check

Author Response: The ADP values for hydrogen atoms were estimated using SHADE3.

PLAT218_ALERT_3_C Constrained U(ij) Components(s) for H13 . 6 Check

Author Response: The ADP values for hydrogen atoms were estimated using SHADE3.

PLAT218_ALERT_3_C Constrained U(ij) Components(s) for H23 . 6 Check

Author Response: The ADP values for hydrogen atoms were estimated using SHADE3.

PLAT218_ALERT_3_C Constrained U(ij) Components(s) for H8 . 6 Check

Author Response: The ADP values for hydrogen atoms were estimated using SHADE3.

PLAT218_ALERT_3_C Constrained U(ij) Components(s) for H14 . 6 Check

Author Response: The ADP values for hydrogen atoms were estimated using SHADE3.

PLAT218_ALERT_3_C Constrained U(ij) Components(s) for H17 . 6 Check

Author Response: The ADP values for hydrogen atoms were estimated using SHADE3.

```
PLAT218_ALERT_3_C Constrained U(ij) Components(s) for H18        .          6 Check
```

Author Response: The ADP values for hydrogen atoms were estimated using SHADE3.

```
PLAT218_ALERT_3_C Constrained U(ij) Components(s) for H20        .          6 Check
```

Author Response: The ADP values for hydrogen atoms were estimated using SHADE3.

```
PLAT218_ALERT_3_C Constrained U(ij) Components(s) for H24        .          6 Check
```

Author Response: The ADP values for hydrogen atoms were estimated using SHADE3.

```
PLAT351_ALERT_3_C Long   C-H (X0.96,N1.08A)  C11      - H11      .       1.11 Ang.
```

Author Response: The hydrogen atom positions have been refined with HAR, therefore, in some cases, bond lengths do not correspond to the neutron value.

```
PLAT351_ALERT_3_C Long   C-H (X0.96,N1.08A)  C25      - H25      .       1.11 Ang.
```

Author Response: The hydrogen atom positions have been refined with HAR, therefore, in some cases, bond lengths do not correspond to the neutron value.

```
PLAT351_ALERT_3_C Long   C-H (X0.96,N1.08A)  C26      - H26      .       1.11 Ang.
```

Author Response: The hydrogen atom positions have been refined with HAR, therefore, in some cases, bond lengths do not correspond to the neutron value.

```
PLAT911_ALERT_3_C Missing FCF Refl Between Thmin & STh/L=    0.600        117 Report
```

Author Response: Determining the exposure time on Pilatus 1M CdTe is problematic. One of runs had to be removed from data processing due to improper exposure time.

Alert level G

```
ABSMU01_ALERT_1_G  Calculation of _exptl_absorpt_correction_mu
                not performed for this radiation type.
PLAT019_ALERT_1_G _diffrn_measured_fraction_theta_full/*_max < 1.0      0.985 Report
PLAT092_ALERT_4_G Check: Wavelength Given is not Cu,Ga,Mo,Ag,In Ka    0.24820 Ang.
PLAT164_ALERT_4_G Nr. of Refined C-H H-Atoms in Heavy-Atom Struct.         19 Note
PLAT232_ALERT_2_G Hirshfeld Test Diff (M-X)  Au1      --C1       .        6.0 s.u.
PLAT371_ALERT_2_G Long   C(sp2)-C(sp1) Bond  C2       - C3       .       1.44 Ang.
PLAT434_ALERT_2_G Short Inter HL..HL Contact Cl1      ..Cl1      .       3.26 Ang.
                                         -x,-y,1-z  =      3_556 Check
PLAT802_ALERT_4_G CIF Input Record(s) with more than 80 Characters          1 Info
```

```
PLAT872_ALERT_4_G ALERTS Related to Anharmonic Refine   Suppressed          ! Info
PLAT883_ALERT_1_G No Info/Value for _atom_sites_solution_primary .     Please Do !
PLAT910_ALERT_3_G Missing # of FCF Reflection(s) Below Theta(Min).          1 Note
PLAT912_ALERT_4_G Missing # of FCF Reflections Above STh/L=  0.600        216 Note
PLAT933_ALERT_2_G Number of HKL-OMIT Records in Embedded .res File         31 Note
PLAT960_ALERT_3_G Number of Intensities with I  <   - 2*sig(I) ...          4 Check
PLAT978_ALERT_2_G Number C-C Bonds with Positive Residual Density.         10 Info
PLAT979_ALERT_1_G NoSpherA2 Scattering Factors Used .............     Please Note
```

```
   1 ALERT level A = Most likely a serious problem - resolve or explain
   1 ALERT level B = A potentially serious problem, consider carefully
  23 ALERT level C = Check. Ensure it is not caused by an omission or oversight
  16 ALERT level G = General information/check it is not something unexpected

   5 ALERT type 1 CIF construction/syntax error, inconsistent or missing data
   5 ALERT type 2 Indicator that the structure model may be wrong or deficient
  26 ALERT type 3 Indicator that the structure quality may be low
   5 ALERT type 4 Improvement, methodology, query or suggestion
   0 ALERT type 5 Informative message, check
```

It is advisable to attempt to resolve as many as possible of the alerts in all categories. Often the minor alerts point to easily fixed oversights, errors and omissions in your CIF or refinement strategy, so attention to these fine details can be worthwhile. In order to resolve some of the more serious problems it may be necessary to carry out additional measurements or structure refinements. However, the purpose of your study may justify the reported deviations and the more serious of these should normally be commented upon in the discussion or experimental section of a paper or in the "special_details" fields of the CIF. checkCIF was carefully designed to identify outliers and unusual parameters, but every test has its limitations and alerts that are not important in a particular case may appear. Conversely, the absence of alerts does not guarantee there are no aspects of the results needing attention. It is up to the individual to critically assess their own results and, if necessary, seek expert advice.

Publication of your CIF in IUCr journals

A basic structural check has been run on your CIF. These basic checks will be run on all CIFs submitted for publication in IUCr journals (*Acta Crystallographica*, *Journal of Applied Crystallography*, *Journal of Synchrotron Radiation*); however, if you intend to submit to *Acta Crystallographica Section C* or *E* or *IUCrData*, you should make sure that full publication checks are run on the final version of your CIF prior to submission.

Publication of your CIF in other journals

Please refer to the *Notes for Authors* of the relevant journal for any special instructions relating to CIF submission.

PLATON version of 20/01/2022; check.def file version of 19/01/2022

Datablock mm_apex3 - ellipsoid plot

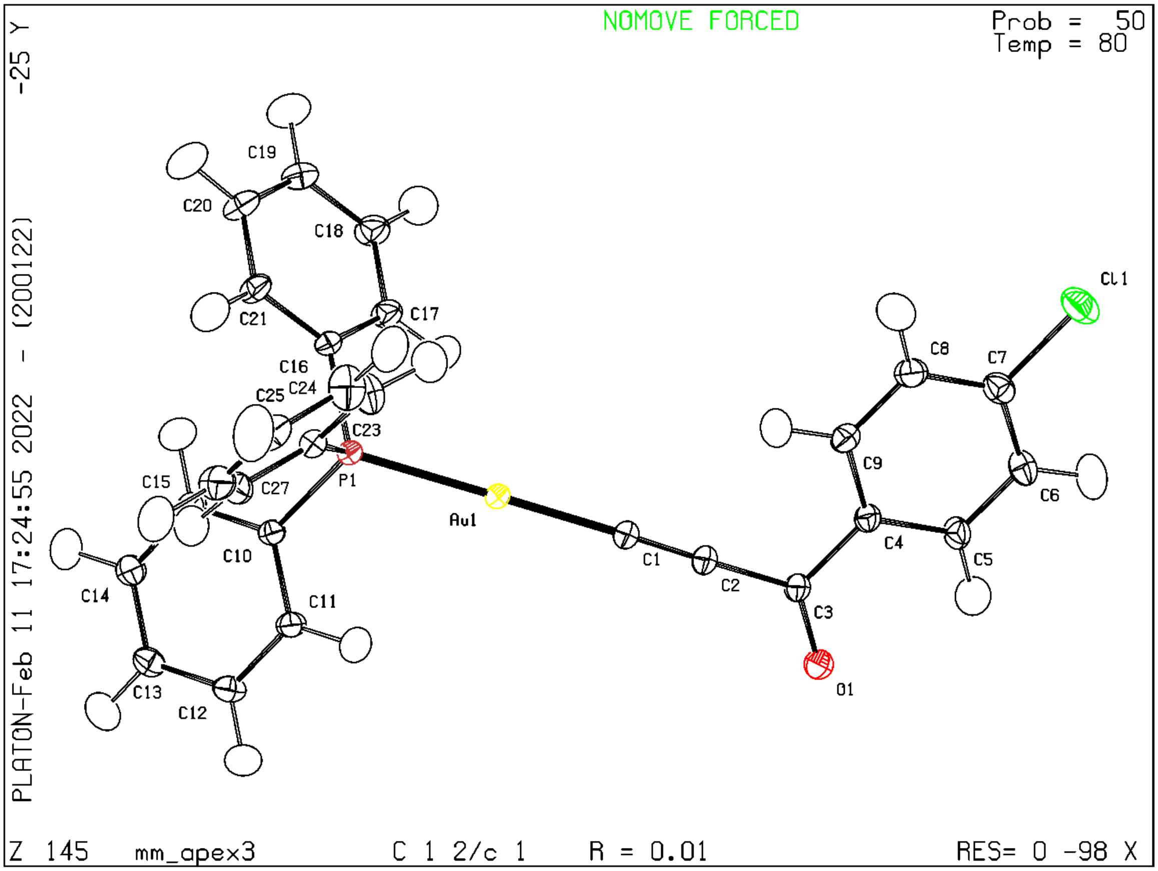

Supplement: Supplementary file 3 [file m-09-00497-sup3.zip › CIFs_with_check_cif/rks_anh_rel_shade_checkcif.pdf]
